# The Yellow Fever Vaccine Journey: Milestones and Future Directions

**DOI:** 10.3390/vaccines14010065

**Published:** 2026-01-05

**Authors:** Shriyansh Srivastava, Nandani Jayaswal, Pranav Gupta, Sathvik Belagodu Sridhar, Pooja Jaiswal, Mohd. Tariq, G. S. N. Koteswara Rao, Aroop Mohanty, Sanjit Sah, Rachana Mehta, Juan Pablo Hernández-Ovalle, Jaime D. Acosta-España, Lysien Zambrano, Alfonso J. Rodriguez-Morales

**Affiliations:** 1 School of Medical and Allied Sciences, Galgotias University, Greater Noida 203201, Uttar Pradesh, India; shriyanshsrivastav@gmail.com (S.S.); pranavgupta123@gmail.com (P.G.); 2 Faculty of Pharmaceutical Sciences, Mahayogi Gorakhnath University Gorakhpur, Gorakhpur 273007, Uttar Pradesh, India; nandani.jaiswal123@gmail.com (N.J.); pj143710@gmail.com (P.J.); 3 Department of Clinical Pharmacy and Pharmacology, RAK College of Pharmacy, RAK, Medical and Health Sciences University, Ras Al Khaimah 71705, United Arab Emirates; sathvik@rakmhu.ac.ae; 4 Department of Academics, Sumandeep Vidyapeeth (Deemed to be University), Vadodara 391760, Gujarat, India; tariq.du14@gmail.com; 5 Department of Biotechnology, Graphic Era (Deemed to be University), Dehradun 248002, Uttarakhand, India; 6 Shobhaben Pratapbhai Patel School of Pharmacy and Technology Management, SVKM’s NMIMS, Mumbai 400056, Maharashtra, India; drgsnkrao@gmail.com; 7 Department of Microbiology, AIIMS Gorakhpur, Gorakhpur 273008, Uttar Pradesh, India; aroopmohanty7785@yahoo.com; 8 Department of Pediatrics, Dr. D.Y. Patil Medical College, Hospital and Research Centre, Dr. D.Y. Patil Vidyapeeth (Deemed-to-be-University), Pimpri, Pune 411018, Maharashtra, India; sanjitsah101@gmail.com; 9 Department of Public Health Dentistry, Dr. D.Y. Patil Dental College and Hospital, Dr. D.Y. Patil Vidyapeeth, Pune 411018, Maharashtra, India; 10 Department of Medicine, Korea University, Seoul 02481, Republic of Korea; 11 Dr. Lal PathLabs Nepal, Chandol, Kathmandu 44600, Nepal; mehtarachana89@gmail.com; 12 Clinical Microbiology, RDC, Manav Rachna International Institute of Research and Studies, Faridabad 121004, Haryana, India; 13 Program of Family Medicine Specialization, Faculty of Medicine, Fundación Universitaria Autónoma de las Américas-Institución Universitaria Visión de las Américas, Pereira 660007, Risaralda, Colombia; juan.hernandez@uam.edu.co; 14 Health Sciences Faculty, Universidad Internacional SEK (UISEK), Quito 170120, Ecuador; jaime.acosta@uisek.edu.ec; 15 School of Medicine, Pontificia Universidad Católica del Ecuador, Quito 170120, Ecuador; 16 Institute of Microbiology, Friedrich Schiller University Jena, 07737 Jena, Germany; 17 Research Group of Emerging and Neglected Diseases, Ecoepidemiology and Biodiversity, Health Sciences Faculty, Universidad Internacional SEK (UISEK), Quito 170120, Ecuador; 18 Department of Morphological Sciences, School of Medical Sciences, Universidad Nacional Autónoma de Honduras, Tegucigalpa 11101, Honduras; lysien.zambrano@unah.edu.hn; 19 Faculty of Health Sciences, Universidad Científica del Sur, Lima 15067, Peru; 20 Grupo de Investigación Biomedicina, Faculty of Medicine, Fundación Universitaria Autónoma de las Américas-Institución Universitaria Visión de las Américas, Pereira 660003, Risaralda, Colombia

**Keywords:** vaccinology, immunopreparedness, epidemiosurveillance, attenuation, reemergence

## Abstract

Yellow fever, a mosquito-borne viral hemorrhagic disease, remains a significant public health concern in endemic regions of Africa and South America. The development of the yellow fever vaccine marked a milestone in virology and immunization. In the 1930s, Max Theiler created the 17D live-attenuated vaccine, a breakthrough that has achieved global recognition and continues to underpin prevention strategies. This review outlines the historical evolution of the yellow fever vaccine, highlighting pivotal scientific advances, technological innovations, and global eradication initiatives. It examines the current landscape of immunization, focusing on the World Health Organization’s Eliminate Yellow Fever Epidemics (EYE) strategy, ongoing efforts to address vaccine supply constraints, and persistent surveillance gaps. Future directions in vaccine development, including next-generation platforms and improved delivery systems, are also discussed, alongside the need for sustained research investment and international collaboration. As yellow fever emerges in previously non-endemic areas due to climate change and globalization, strengthening vaccination programs remains critical to preventing outbreaks and ensuring effective disease control.

## 1. Introduction

Yellow fever is endemic to or has endemic zones in thirteen Latin American countries [1] and thirty-four African countries as of 2023 [2]. A safe and inexpensive vaccine can prevent yellow fever [3]. The yellow fever vaccination requires only one shot to protect against the disease for life [4,5].

Yellow fever’s first appearance in the Americas is uncertain due to a lack of early medical records and difficulty in retrospective diagnosis in 1648 [6]. Yellow fever (YF) is believed to have originated in Africa, yet the first documented case in the Americas apparently occurred in that year in Yucatán, Mexico. Over subsequent centuries, the disease spread widely across tropical Africa and the Americas via maritime and overland trade routes, severely affecting major port cities. Montevideo (Uruguay), Tocopilla (Chile), and Quebec (Canada) experienced devastating outbreaks linked to international commerce. In the United States, New York City endured at least 25 epidemics between 1668 and 1870, while numerous other cities were also heavily affected. In Europe, YF posed a significant threat as well; in 1821, Barcelona faced a severe epidemic, and the earliest recorded European case was reported in Cádiz, Spain. Historical outbreaks have been documented by year and geographic region, illustrating the extensive global reach of the disease [7]. In 2013, an estimated 130,000 cases of yellow fever were reported in Africa [8]. The tropical regions of ten Latin American and thirty-one African nations are endemic to the flavivirus that causes yellow fever (YF). In Africa, the disease is transmitted by the *Aedes africanus* mosquito in the sylvatic settings, while in South America, it is spread mainly by the *Haemagogus* and *Sabethes* species [2]. The most common hosts for this pathogen are monkeys [9]. The YFV-urban transmission cycle was significantly influenced by the mosquito *Aedes aegypti*, which successfully expanded its geographic distribution due to its ability to breed in small amounts of water, produce desiccation-resistant eggs, and prefer human interaction [10].

*Aedes aegypti* has expanded its geographic range due to multiple ecological drivers, including rapid urbanization with inadequate water and waste management, increasing globalization and human mobility, and climate change that favors vector survival in new areas [11]. In addition, limited or inconsistent vector control programs and the proliferation of artificial water containers in urban environments have facilitated its establishment in regions previously unsuitable for transmission. These factors together explain the continued spread and resilience of *Aedes aegypti* populations across tropical and subtropical regions.

**Figure 1:**
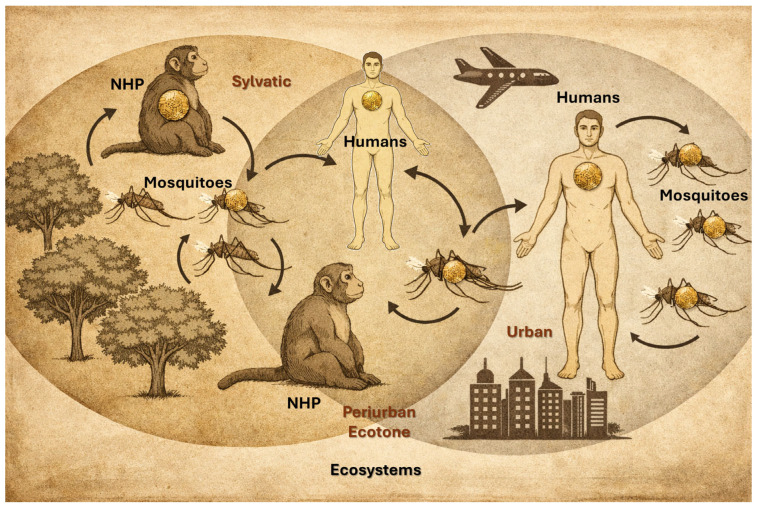
Yellow fever main cycles. Adapted from [13].

The yellow fever virus is maintained in nature through three distinct transmission cycles. The sylvatic (jungle) cycle involves transmission between non-human primates (NHPs) and sylvatic mosquitoes, with humans infected incidentally when entering forested areas (Figure 1) [12]. The intermediate (savannah) cycle, unique to Africa, occurs in rural or peri-urban settings where both humans and NHPs are bitten by semi-domestic mosquitoes, leading to localized outbreaks. In contrast, the urban cycle is sustained by human–mosquito–human transmission, primarily mediated by *Aedes aegypti*, and is responsible for large explosive epidemics in densely populated areas. Understanding these cycles is critical for assessing outbreak risk, implementing vaccination strategies, and guiding vector control efforts [13].

NHPs are key to surveillance, serving as an early warning system for yellow fever, as primate die-offs usually precede human cases. It enables quicker vaccination and vector control, proving more timely than human case tracking. However, its reliability depends on reporting, diagnostics, and coverage, making it best used as a complement to human surveillance [13,14].

In Africa, yellow fever has a relatively low case-fatality rate. The layered transmission ecology, coupled with overlapping environmental and social factors, contributes to more frequent and widespread outbreaks on the African continent [15]. Yellow fever is a severe acute illness characterized by fever, nausea, vomiting, and hepatitis, with 20–60% of cases resulting in death [15].

The 1930s development of live, attenuated YF vaccines and their widespread deployment in the 1940s contributed to the decline of the disease; however, periodic YF activity persists in endemic regions [16]. The 17D strain, a safe and effective vaccination, was produced in 1936. YF vaccines are produced by inoculating viral seeds into chicken embryos and subsequently collecting the infected embryos according to the World Health Organization (WHO) regulations [17]. Since 1970, vaccine coverage has improved, but gaps persist in areas with a high risk of yellow fever. An estimated 393–472.9 million people still need vaccination to reach at least 80% of the minimum coverage threshold recommended by the WHO, compared to 66–76% in 1970 [18]. For example, in Africa and the Americas over the last two decades, YF vaccine coverage rates have been far from optimal (Table 1). To achieve the WHO-recommended threshold of 80% vaccination coverage, multifaceted strategies are essential. Expanding routine immunization is the cornerstone, ensuring that the YF vaccine is systematically included in national immunization schedules, particularly in endemic countries. Mass preventive and catch-up campaigns should target unvaccinated populations, including migrants, rural communities, and those in conflict-affected or remote regions where access is limited. Strengthening surveillance systems and linking them with outbreak response mechanisms can rapidly identify immunity gaps and trigger targeted vaccination drives. Innovative approaches such as fractional-dose vaccination, already used successfully in emergency settings, may help extend vaccine supplies during shortages. Furthermore, improving community engagement and health education is key to overcoming vaccine hesitancy, while cross-border coordination in endemic regions helps prevent transnational spread. Finally, robust international support through the Eliminate YF Epidemics (EYE) strategy, combined with adequate funding and supply chain management, will be critical to closing coverage gaps and ensuring sustainable protection (https://www.who.int/initiatives/eye-strategy) (accessed on 1 December 2025).

Although vaccination remains underutilized in some settings, its critical role in prevention is reinforced by joint efforts between the Global Alliance for Vaccines and Immunization (Gavi) and the World Health Organization (WHO) to strengthen laboratory-based detection and ensure effective vaccine delivery. While current vaccines are not without limitations, they remain highly effective in mitigating and preventing yellow fever epidemics [19]. Between 2000 and 2006, the majority of side effects reported to the Vaccine Adverse Event Reporting System (VAERS) were mild and resolved spontaneously. Common symptoms included fever, pain, itching, headache, injection-site redness, urticaria, and rash. Like with other vaccines, more adverse events and local inflammatory events were recorded by women [20]. This review highlights the history, development, immunology, safety, and prospects of the yellow fever vaccine, and educates readers about its multifaceted nature and potential advancements.

## 2. Milestones in Yellow Fever Vaccine Development

The most significant milestone in the prevention of yellow fever was the development of the live-attenuated 17D vaccine by Max Theiler in 1936, for which he received the Nobel Prize in Physiology or Medicine in 1951. Since its introduction, the 17D vaccine has remained the cornerstone of yellow fever prevention and control worldwide. Large-scale deployment of this vaccine during the 1940s contributed substantially to the decline of urban yellow fever outbreaks in the Americas and Africa [21].

The 17D vaccine is produced using embryonated chicken eggs, a manufacturing process that has remained essentially unchanged since the 1940s. Each dose contains between 10^4^ and 10^6^ plaque-forming units of virus, and six major manufacturers currently supply 30–60 million doses annually under the supervision of the World Health Organization (WHO) [22]. By 2023, more than 500 million people worldwide had received the vaccine, confirming its central role in global immunization programs. Initially, WHO recommended booster doses every 10 years, but current evidence supports long-lasting, possibly lifelong protection after a single dose [3,23].

A booster dose of the yellow fever 17D vaccine is not recommended for the general population, as multiple lines of evidence indicate that a single dose induces long-lasting, likely lifelong immunity in most individuals. After vaccination, neutralizing antibodies typically persist for decades, and CD8^+^ T cells and memory B cells provide durable immune memory that can rapidly respond upon re-exposure—even if antibody titers wane over time. Extensive cohort studies and outbreak investigations have consistently demonstrated that vaccine failures are exceedingly rare, even decades after primary immunization [24,25,26].

In 2013, the World Health Organization (WHO) concluded that a single dose provides lifelong protection and revised the International Health Regulations accordingly, no longer requiring boosters for international travel. Boosters are recommended only for specific high-risk groups (e.g., people vaccinated while immunocompromised, young children vaccinated before age 2, or individuals with HIV) because they may elicit lower or shorter-lived immune responses (https://www.who.int/news-room/fact-sheets/detail/yellow-fever, accessed on 1 December 2025).

Although the complete molecular basis of 17D attenuation remains incompletely elucidated, multiple genomic regions and specific mutations—particularly within the envelope (E) protein, NS2A, and other nonstructural genes—have been identified as key contributors to its attenuated phenotype (Table 2). Nevertheless, its safety and efficacy have made it a model for live-attenuated viral vaccines. Research on next-generation vaccines—including inactivated, recombinant, and chimeric platforms—has emerged to address supply shortages and broaden immunization strategies. These candidate vaccines, however, are better discussed in the context of future directions [27].

A study analyzed CD8^+^ T cells responding to live yellow fever virus and smallpox vaccines. Results showed a significant primary effector response, high specificity, and memory differentiation. Vaccines also generated virus-specific CD8^+^ T cells, which decreased by more than 90% and transformed into long-lasting memory cells [28]. Live-attenuated viral vaccines, exemplified by the yellow fever virus strain 17D, are both safe and efficacious yet possess complex attenuation mechanisms. This work examines the 17D attenuation mechanism and determines its resistance to ribavirin, suggesting a minimal likelihood of reversion or mutation, hence preserving a stable genotype despite external influences [29]. The genome of the ARILVAX live-attenuated yellow fever (YF) 17D vaccine was analyzed by consensus sequencing, revealing 12 nucleotide variants. These differences suggest a heterogeneous population, with some individuals exhibiting quasispecies. Some nts differed from some strains but coincided with others, possibly due to the consensus sequencing approach. Most heterogeneities were silent, and other YF 17D vaccines need further examination using consensus sequencing [30]. Although these vaccinations are effective against viral infections, the mechanisms underlying the attenuation of live attenuated viruses remain unknown. Understanding the molecular mechanisms underlying attenuation is crucial for the use of the 17D live-attenuated vaccine strain to deliver heterologous antigens. Type II interferon protected mice from mortality and restricted viral replication to lymphoid organs, inhibiting 17D replication by 1–2 days post-infection in murine models. In vitro, IFN-γ treatment inhibited 17D replication more effectively than WT YFV [31,32].

The Baculovirus Expression Vector System (BEVS) production technology has reached a level of maturity for commercial manufacture, as evidenced by the approval of vaccines such as CERVARIX. BEVS is a recombinant protein production technology that uses genetically engineered baculoviruses to deliver and express foreign genes in insect host cells, enabling the efficient manufacture of complex biological products, particularly recombinant vaccine antigens [33]. Insect cell-based products could be developed more quickly in the future due to reduced uncertainty during product development. This technology’s “plug and play” feature may make it sustainable in low-resource settings by enabling mass production of vaccines tailored to individual needs [34]. Vaccines have evolved due to factors like safety, tolerability, and potency. Improved vaccines have necessitated more stringent manufacturing processes, whereas older vaccines, such as influenza vaccines, are still produced using outdated technologies. Modern manufacturing processes are essential for producing vaccines that are equivalent to or superior to their predecessors, while new technologies may be necessary to develop innovative vaccines. Despite these advancements, challenges persist, including the need to adapt manufacturing procedures, address regulatory issues, and manage rising costs [35]. This study employed a regression model, using serological data from 11 countries, to estimate the yellow fever burden across 34 African nations.

Vaccinations administered since 2005 are estimated to prevent 3–6 million deaths over the lifetimes of those immunized. The most significant impact is expected to be the disruption of both sylvatic and urban transmission cycles. Projections indicate that allocating 37.7 million doses annually for preventive mass vaccination campaigns (PMVCs) between 2018 and 2026 could avert approximately 9.89 million yellow fever infections and substantially reduce the risk of global spread. Addressing the potential shift from sylvatic to urban human-to-human transmission, the study underscores the critical role of achieving high vaccination coverage in preventing large-scale outbreaks [36,37]. Over the past four decades, substantial progress has been achieved in vaccine deployment, driven by sustained global political commitment, effective program management, and rigorous quality control measures. Although significant challenges persist in low-income countries, vaccination services are now reaching unprecedented numbers of individuals. This success underscores the need for sustained investment and additional resources to maintain and expand coverage [38].

## 3. Yellow Fever Vaccine Immunology

Mosquitoes transmit the viral illness known as yellow fever, and one of the most effective ways to prevent its spread has been through vaccination. The 17D live-attenuated yellow fever vaccine is used to prevent the disease. The 17D vaccine, a safe and effective live virus vaccine, is derived from chick embryos inoculated with a seed virus. It forms the basis for the 17D-204 and 17DD lineages, with 99.9% sequence homology [39]. Nucleotide sequence analysis is used to determine YFV diversity, and evolutionary rates are constant across regions. There can be as much as a 16% disparity between genotypes from South America and Africa [40]. Results from the 36 trials demonstrate that the yellow fever vaccine is highly effective in inducing an immune response and providing lifelong protection against the virus with a single dose. The following groups are given extra care: those with HIV, very young children, pregnant women, and those with severe malnutrition; hence, a booster dosage is not necessary [41]. A single-vaccination strategy based on YF-specific CD8^+^ T-cell profiles and neutralizing antibodies is recommended in yellow fever vaccination guidelines. It is possible to achieve long-term immunity with a single vaccination if there is an adequate supply of neutralizing antibodies and a functionally competent pool of memory T-cells [26].

The use of bioinformatics-predicted antigens in yellow fever vaccine development offers a promising avenue for improving both efficacy and safety. Computational analyses enable the identification of conserved, immunodominant epitopes within the yellow fever virus (YFV) genome, with particular focus on structural proteins such as the envelope (E) protein and non-structural proteins like NS1 [42,43]. These in silico methods enable the prediction of B-cell and T-cell epitopes with high immunogenic potential, reducing the reliance on traditional empirical approaches. Epitope-based vaccines can be designed to elicit targeted immune responses while minimizing adverse effects associated with live-attenuated vaccines, such as the 17D strain [44]. This strategy holds significant promise for next-generation vaccines, particularly for populations with contraindications to live vaccines and for rapid responses to future outbreaks [45,46]. However, there are no yet epitope-based YF vaccines in human efficacy trials, and the most advanced non-live approaches remain preclinical or in early clinical trials. So replacement of 17D in control programs is unlikely for years.

Accurate prediction of epitopes remains essential for advancing rational vaccine design against flaviviruses, including the YFV. Among available tools, the NetMHCpan and NetMHCIIpan suites (currently versions 4.1 and 4.0/4.1) are considered gold standards for T-cell epitope prediction, as they integrate peptide–MHC binding affinity with data from mass spectrometry–eluted ligands, achieving high predictive performance across a broad spectrum of HLA alleles. Similarly, MHCflurry 2.0 improves accuracy for class I epitopes by incorporating antigen processing (proteasomal cleavage and TAP transport), which is crucial for predicting peptides presented on the cell surface [47].

For B-cell epitopes, tools such as BepiPred-2.0 and EpiDope have demonstrated reliable performance in identifying linear epitopes from protein sequences. In contrast, structure-based methods such as DiscoTope 2.0, SEPPA 3.0, and the more recent SEMA 2.0 enhance the detection of conformational epitopes by leveraging 3D structural data. These approaches are particularly relevant for flaviviruses, where neutralizing antibodies often target conformational epitopes in the envelope (E) protein and nonstructural protein 1 (NS1) [48].

Collectively, the integration of multiple computational approaches—sequence-based and structure-based—has emerged as the most robust strategy for epitope mapping in YFV and related flaviviruses. This is not only critical for identifying promising immunogenic regions but also for guiding next-generation vaccine platforms and immunotherapeutic development [49].

Epitope-based vaccines have the potential to provide cross-protection across yellow fever virus (YFV) genotypes from Africa and South America because many of the most protective antibody targets on the viral envelope (E) protein are conserved. The licensed 17D vaccine, derived from a West African strain, demonstrates this principle by providing global protection, including against South American outbreaks. Structural studies show that neutralizing epitopes in domains I and II of E are critical and relatively stable. In contrast, broad and durable CD4^+^ and CD8^+^ T-cell epitopes—often conserved across lineages—further contribute to long-term protection. This evidence supports the feasibility of designing vaccines based on conserved B- and T-cell epitopes to achieve cross-genotype immunity [42,50].

However, studies also indicate that some South American isolates are less sensitive to vaccine-induced antibodies, suggesting genotype-specific differences that could limit protection if vaccines focus too narrowly on single epitopes [51,52]. To overcome this, next-generation epitope-based vaccines should employ structure-guided design, presenting conserved E-protein epitopes in a native-like context (e.g., nanoparticles or subviral particles) and incorporating genotype-variant residues through mosaic or multivalent strategies [51,52]. Coupled with conserved, promiscuous T-cell epitopes, such designs could mitigate reduced susceptibility while ensuring durable cellular and humoral responses across regions. This approach balances the proven cross-protection of 17D with the emerging evidence of genotype-specific variation [51,52].

Significant limitations of in silico–predicted antigens in clinical settings arise from the gap between computational predictions and real-world immune responses. Although tools for epitope mapping of B-cell and T-cell responses have become increasingly sophisticated, they rely on algorithms trained on limited datasets and simplified assumptions regarding antigen processing, HLA restriction, and epitope presentation. Consequently, predictions may generate false positives (epitopes that fail to elicit immune responses) or false negatives (failure to identify truly immunogenic regions). Significantly, T-cell epitopes are not restricted to structural or classically antigenic proteins; robust CD4^+^ and CD8^+^ T-cell responses have been shown to target non-structural (NS) proteins, which are often underrepresented or excluded from epitope-based vaccine design strategies. This limitation may result in the omission of highly conserved and immunodominant T-cell targets that contribute significantly to protective cellular immunity [53,54]. Moreover, many computational approaches prioritize linear peptide epitopes. In contrast, several neutralizing antibody targets are conformational and discontinuous, requiring correct protein folding and post-translational processing—features that cannot be fully captured by in silico models alone [53].

Another critical limitation is the lack of integration of host-specific factors, including HLA polymorphisms, T-cell receptor repertoires, and prior immunity, which strongly influence immunogenicity. In silico predictions also rarely account for post-translational modifications (e.g., glycosylation in flaviviruses like YFV) that affect antigenicity. Significantly, even when predicted epitopes bind MHC molecules with high affinity, they may not elicit protective immunity or may even induce tolerance or off-target effects. Thus, while computational approaches accelerate antigen discovery, experimental validation in vitro and in vivo remains indispensable before clinical translation [55,56].

### 3.1. Mechanisms of Immune Response to the Yellow Fever Virus

**Figure 2:**
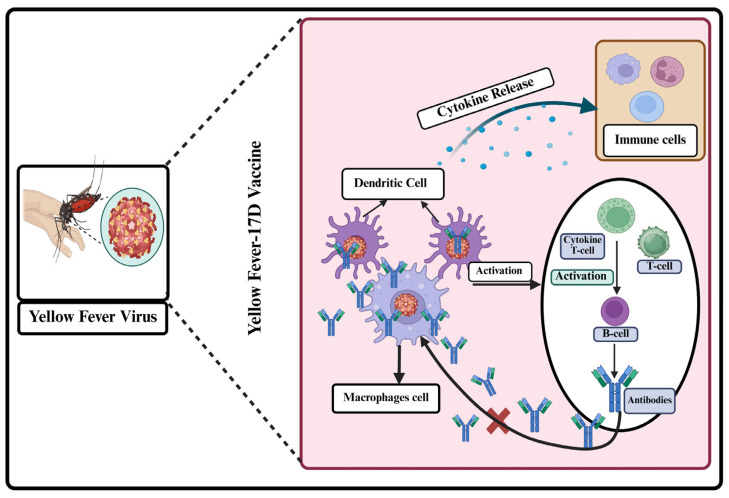
Yellow fever virus interacts with the immune response at multiple stages, from initial recognition to adaptive immune activation.

The immune response to the yellow fever virus involves the cooperation of both innate and adaptive immune systems to identify, combat, and eradicate the virus. Figure 2 illustrates the immune response to the yellow fever virus as a coordinated defense mechanism involving both innate and adaptive immunity. It begins with innate recognition, cytokine release, and the activation of natural killer cells, followed by a T-cell response and the production of neutralizing antibodies.

#### 3.1.1. Innate Immune Responses to Vaccination with 17D

NK cell status after vaccination shows increased expression of TLR-3 and nine markers [26]. The study explores the immune response to yellow fever vaccination, revealing the significant role of innate immunity. It highlights increased TNF+ neutrophils, IFN+ NK cells, and IL-10+ monocytes, and identifies primary sources of pro- and anti-inflammatory cytokines that contribute to protective immunity [57]. A 17-day yellow fever vaccine elicited an innate immune response in peripheral blood, as evidenced by elevated monocyte counts, NK cell subpopulations, granulocyte activation, FcR and IL-10R expression, and results reported in [28]. Children vaccinated with either YF-17DD or YF-17D-231/77 yellow fever strain exhibited a well-regulated inflammatory and regulatory immune response, as indicated by the study’s analysis of cytokine-mediated immune responses [58]. Attenuated live yellow fever virus 17D primary immunization stimulates innate immunity, decreases peripheral blood T and B cells, and promotes robust adaptive immunity, according to the study [59]. Live attenuated yellow fever vaccines are effective and safe worldwide. PRNT antibodies exhibit a consistent innate and adaptive immune response, a proinflammatory and regulatory profile, and a polarized regulatory reaction, with PV-PRNTMEDIUM1 serving as the hallmark [60].

Recent studies highlight that increased IL-10^+^ monocytes following yellow fever vaccination play a dual role. While traditionally considered anti-inflammatory, these cells also contribute to immune regulation by balancing pro-inflammatory responses and preventing excessive immunopathology. This regulatory function may be crucial for the controlled induction of adaptive immunity without triggering damaging inflammation. Additionally, the upregulation of Fc receptors (FcγRs) on innate cells suggests that the vaccine promotes a form of “trained immunity” or innate memory, enhancing the capacity of monocytes and dendritic cells to respond more effectively to subsequent antigen exposure. This phenomenon may underpin the robust and durable adaptive immune responses observed with the 17D vaccine [61,62].

#### 3.1.2. Adaptive Immune Response

The adaptive immune response, including cellular and humoral immunity, becomes prominent within a few days after infection or vaccination.

(a) Humoral (antibody) response

In a trial conducted in locations without YFV circulation, 288 children and adults were studied to determine the effect of age and pre-existing flavivirus humoral immunity on vaccination immunity against 17DD-YF [63]. A Swiss experiment evaluated the efficacy of the live-attenuated yellow fever vaccine YF-17D in both the first and subsequent doses. Because baseline neutralizing antibodies impacted both the immunological response and the multiplication of the vaccine virus, the booster dose reduced the risk of adverse effects [64]. A Special Immunizations Program study found that re-vaccination increases antibody levels in patients with low pre-vaccination serologies, and booster vaccination should be considered if persistently at high risk [65]. This study examines the effectiveness of the 17D live-attenuated yellow fever vaccine in individuals with compromised immune systems. They found a memory-like phenotype that is linked to good expansion upon re-encounter with the antigen [66]. The 17D vaccine induces early and viremia-dependent immune system involvement, particularly by CD8^+^ T cells, and efficient neutralizing antibody production. The study found a significant increase in CD8^+^ and CD4^+^ T cells, paralleling viremia, especially in first-time vaccine recipients [67].

In parallel, IFN-γ secretion by activated T cells and NK cells is central to controlling yellow fever virus replication. IFN-γ enhances the antiviral state of infected and neighboring cells by upregulating interferon-stimulated genes, thereby restricting viral spread. This mechanism is particularly effective against the attenuated 17D strain, where IFN-γ–mediated responses further suppress viral replication compared to wild-type strains, contributing to the exceptional safety and immunogenicity profile of the vaccine [68,69].

Regarding correlates of protection, the most widely accepted threshold for neutralizing antibodies is a 50% plaque-reduction neutralization test titer of 1:10, which has been consistently used as the benchmark in vaccine efficacy studies. Interestingly, booster doses do not uniformly enhance immunity; in individuals with high baseline antibody titers, boosters often yield a reduced response. This effect is thought to result from immune interference or antigenic competition, where pre-existing antibodies neutralize the vaccine virus before it can effectively restimulate adaptive responses. Moreover, host genetics also appears to play a role: particular HLA class I alleles have been associated with differences in the magnitude and quality of CD8^+^ T-cell memory responses, suggesting that vaccine immunogenicity may be partly shaped by host genetic background [26,70].

(b) Cellular response

The cellular immune response involves T cells attacking infected cells directly to combat the yellow fever virus. A study on yellow fever vaccination responses identified 92 and 50 epitopes, with many causing strong immunodominant responses, providing broad coverage for academic, diagnostic, and therapeutic purposes [71]. Serious consequences from the yellow fever virus (YFV) can be prevented with vaccination. The 17D YFV strain is an excellent resource for investigating human immunity, as it elicits strong T-cell responses and neutralizing antibodies. Researchers used HLA-DR tetramers to investigate how CD4 T cells from vaccinated individuals responded to YFV. According to the results, YFV-specific T cells remain in the body for up to two weeks following vaccination, with frequencies ranging from zero to one hundred cells per million [72]. High-throughput single B-cell cloning is used in this study to track how individuals’ B cells respond to the 17D yellow fever vaccine. The study found that both conventional and switched immunoglobulin MBC groups influenced B-cell reactivity. Affinity maturation persisted for 6–9 months after vaccination, suggesting that germinal center activity persisted. B-cell responses can be better understood with the help of the results [73]. Two distinct antigen formulations based on nonviral DNA for attenuated yellow fever (YF) virus are presented in this study. The full-length envelope protein and the lysosomal-associated membrane protein signal, LAMP-1, were combined in the pL/YFE formulation so that it could elicit robust T-cell responses against nearly all of the epitopes produced by the YF 17DD vaccination. Overall, the pL/YFE formulation was more effective than the 17DD vaccine, as it produced higher titers of anti-YF neutralizing antibodies. Mice subjected to intracerebral inoculation with the YF virus exhibited total resistance following administration [74]. Early gene signatures that predict immune responses in patients immunized with the yellow fever vaccine YF-17D were elucidated using a systems biology approach. Scientists were able to associate and anticipate YF-17D CD8^+^ T cell responses with 90% accuracy using a gene profile that included C1qB and TNFRS17 [75]. The study analyzes CD8^+^ T cells responding to live yellow fever and smallpox vaccines. Results show a significant primary effector response, high specificity, and memory differentiation. The vaccines also produce virus-specific CD8^+^ T cells, which are functional and distinct from human CD8^+^ T cells specific for persistent viruses [76].

Cell-mediated immunity plays a crucial role in the long-term protection induced by the 17D yellow fever vaccine. CD8^+^ T cells contribute significantly to durable immunity by recognizing and eliminating infected host cells and by producing effector cytokines that sustain antiviral responses. These T cells exhibit long-lived memory phenotypes, persisting for decades after vaccination, and provide rapid recall responses upon viral re-exposure [26,77].

### 3.2. Immune Memory and Duration of Protection

Memory B cells can provide more antibody-producing cells and maintain LLPC numbers. CD8^+^ T lymphocytes eliminate infected cells. Studies have used live vaccines to study human immunological responses to viruses, specifically yellow fever and smallpox [78]. The primary objective of studying human CD8^+^ T cells is to characterize their subsets and elucidate the mechanisms underlying their differentiation, particularly in the context of chronic viral infections. These investigations have examined the diverse responses and phenotypic differences between antigen-experienced and naïve CD8^+^ T cells. Findings demonstrate that antigen-experienced CD8^+^ T cells possess broad antigenic specificity and exhibit polyfunctionality, strong proliferative capacity, and sustained long-term persistence [79]. Developing a live-attenuated vaccine, vYF, has demonstrated minimal safety issues and is well-tolerated. It promotes early innate immunity, YFV-specific antibodies, and effective resistance to virulent challenge. This vaccine is designed to address the global shortage of yellow fever vaccinations [80]. Live attenuated yellow fever vaccination provides lifelong protection against yellow fever by inducing a robust CD8^+^ T cell response. An analysis of 41 vaccinees revealed a decline in the frequency of naïve-like YF-specific CD8^+^ T cells over time. Almost all donors had these cells. In contrast to naïve cells from uninfected donors, these cells exhibited characteristics similar to those of the T stem cell–like memory (Tscm) subset. The findings indicate that YF vaccination is the optimal model for investigating memory CD8^+^ T cells with extended half-lives in humans [81]. A meta-analysis of 36 studies from 20 countries found that seroprotection rates after a single yellow fever vaccine were near 100% by 3 months, remaining high in adults for 5–10 years [82]. The study emphasizes the need for booster YFV doses to maintain protective antibody levels, particularly in areas with ongoing epidemics or epizootics, and prioritizes those who have not received vaccination [83]. To establish vaccination requirements, the study examined the geometric mean titers and seropositivity rates of adults vaccinated at various intervals. The study recommended a booster dosage for prolonged protection in infants and toddlers and observed decreased immunogenicity under normal conditions [84].

## 4. Vaccine Safety and Adverse Events

Yellow fever is usually safe and effective (Table 3). Nevertheless, individuals with compromised immune systems may be more susceptible to adverse effects after a yellow fever vaccine than those with healthy immune systems. Replication of the attenuated vaccine strain poses a rare but real danger of severe illness or death [85]. Safe and effective yellow fever (YF) vaccines have been available since the 1930s. However, there are now more precautions and contraindications than ever before, due to the limited number of reports of major adverse events (SAEs). Nearly all (938 out of 1938) recorded adverse events were safety-related (SAEs) between 2007 and 2013. Reporting rates increased with age, with anaphylaxis reporting the highest in 18-year-olds. The observation that the highest rate of anaphylaxis following yellow fever vaccination has been reported among 18-year-olds remains incompletely understood; however, several factors may contribute. This age coincides with the transition from adolescence to adulthood, when immune responsiveness is often at its peak, potentially predisposing individuals to more vigorous hypersensitivity reactions. In addition, many individuals receive their first yellow fever vaccination at this age, for example, before international travel or military service, meaning that any underlying predisposition to vaccine-related allergic reactions (such as egg protein hypersensitivity) is more likely to manifest at this time [86,87,88].

Another possible explanation concerns reporting bias and surveillance: adolescents and young adults are more frequently included in vaccine safety monitoring for travel or academic reasons, which may increase the detection of adverse events relative to older groups. Although anaphylaxis remains rare overall, the age-specific peak at 18 years suggests that both immunological maturity and exposure circumstances converge in this cohort. Nevertheless, further epidemiological and mechanistic studies are needed to clarify whether this pattern is a true biological phenomenon or an artifact of reporting and cohort characteristics [89,90,91]. Continued education for physicians and travelers is crucial for older travelers [92].

**Table 3:** Safety of the yellow fever vaccine and adverse events.

Number	Category	Description	Frequency	Management	Reference
1	Severe and rare adverse events,	YEL-AVD or YEL AND	Rare	Inactivated YF 17D virus	[93]
2	Serious adverse events	Hypersensitivity events, anaphylactic shock, Viscerotropic disease, and neurologic syndrome	25 in 35 people	17D and 17DD yellow fever Vaccine	[94]
3	Allergic Reactions	Anaphylactic reaction	40 in 5,236,820	Yellow fever vaccine	[95]
4	Severe adverse reactions	YEL-AVD, YEL-AEs, and YEL-AND	6 patients	17D-derived yellow fever vaccine	[96]
5	adverse events	Fever, myalgia, and headache	43 in 68 Adult	yellow fever live-attenuated vaccine	[97]

### 4.1. Common Adverse Events: Insights from Post-Marketing Surveillance

Minor adverse effects, including fever and upper respiratory symptoms (e.g., nasal congestion, sore throat), were observed in a systematic assessment of events following yellow fever vaccination in at-risk populations. There were two examples of significant adverse outcomes resulting from maternal-neonate transfer, and older persons were detected in passive monitoring databases as having modest cases of viscerotropic disease, neurotropic disease, and allergy. The number of cases of serious side effects may be lower than the total number of people who got the vaccine [98]. Two cases of hemorrhagic fever have been recorded in Brazil, both linked to the 17DD substrain of the yellow fever vaccine. A 22-year-old African American woman died six days after contracting YF, presenting jaundice, renal failure, and hemorrhagic diathesis; another case included a 5-year-old Caucasian girl who passed away after five days of illness. Both C6/36 cells and nursing mice tested positive for the yellow fever virus [99].

The yellow fever vaccine is generally considered safe, as evidenced by studies using data from the U.S. Department of Defense and the Vaccine Safety Datalink. Nevertheless, rare cases of severe illness and death have been reported following vaccination. Comparative analyses found no significant differences in the incidence of allergic reactions, local reactions, or moderate systemic responses between vaccinated and unvaccinated individuals [100]. Viscerotropic disease, a high-mortality yellow fever vaccine-related disease, has been reported in 26 cases, potentially linked to autoimmune diseases. Bio-Manguinhos/Fiocruz is working on vaccine improvements [101]. Researchers identified 66 studies that met these criteria; 25 employed passive monitoring, 24 active surveillance, and 17 combined the two. No cases of viscerotropic or neurotropic disease were observed in 2,660,929 patients in the general population who were actively monitored. In Switzerland, the United Kingdom, and Australia, a total of 107,621,154 patient data records were sourced from pharmacovigilance databases. Passive surveillance included 94,500,528 individuals, with no serious adverse events proven. Regarding clinical and laboratory follow-up, each country’s database had its own distinct set of regulations, processes, and monitoring instruments.

**Figure 3:**
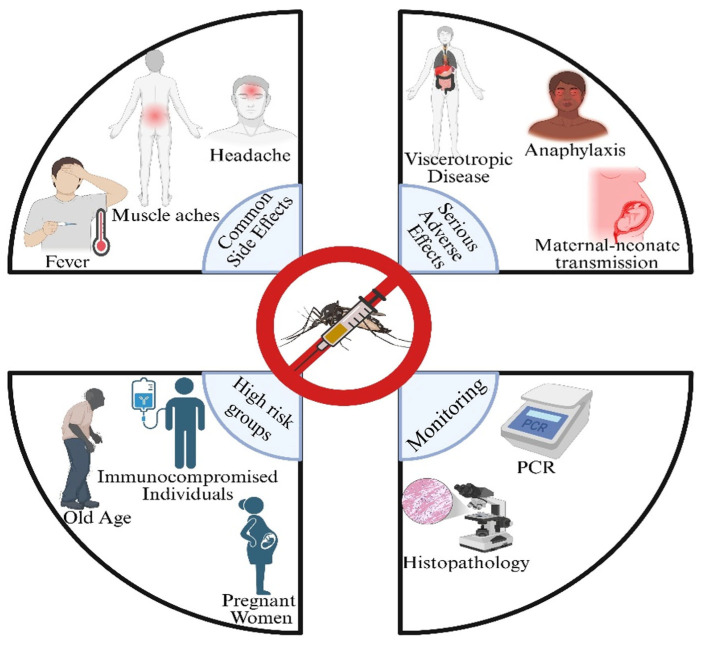
Adverse events & insights from post-marketing surveillance.

Data from Brazil and Australia yield a lower estimate, data from the United States, obtained through VAERS, yield a medium estimate, and data from the United Kingdom and Switzerland yield a higher estimate, according to the pharmacovigilance databases. It is crucial to refine diagnostic techniques, such as PCR amplicon sequencing, pathology, and histology, to determine if the yellow fever vaccine causes serious adverse events [102]. The ARILVAXTM vaccination achieved a higher seroconversion rate (94.9%) than YF-VAX (90.6%), according to a phase III, randomized, double-masked study conducted in northern Peru. The majority of side effects were moderate and resolved spontaneously after vaccination, and both were highly immunogenic. Two YF vaccinations have never been compared in a pediatric population before in a randomized, double-masked study [103] (Figure 3).

At present, there are no validated biomarkers in routine clinical practice that reliably predict a patient’s risk before administration of the yellow fever vaccine. Risk assessment is instead based primarily on demographic and clinical factors. Advanced age, particularly in individuals over 60 years, has been associated with a higher likelihood of serious adverse events such as yellow fever vaccine–associated viscerotropic and neurotropic disease, reflecting age-related immunosenescence and altered immune regulation. Similarly, immunocompromised patients, including those with HIV infection, transplant recipients, or individuals receiving immunosuppressive therapy, exhibit both reduced vaccine immunogenicity and increased susceptibility to complications. Clinical red flags, such as a history of thymus disease, autoimmune conditions, or severe allergies (especially to egg proteins), are also considered necessary when evaluating risk before vaccination [104,105,106].

Emerging research has identified potential immunological and genetic indicators that may contribute to differential vaccine responses, although these remain exploratory. Specific HLA alleles, such as HLA-B07 and HLA-B35, have been implicated in modulating CD8^+^ T-cell memory responses. Additionally, baseline neutralizing antibody titers appear to influence subsequent responses, with high pre-vaccination levels associated with reduced booster effects, possibly due to immune interference. Systems biology approaches, including transcriptomic and cytokine profiling, have also suggested that early innate signatures—such as IL-10–producing monocytes, Fc receptor upregulation, and type I interferon responses—may predict adaptive immunity. However, these findings are confined to the research domain and have not yet been translated into clinical practice. Thus, risk evaluation for yellow fever vaccination currently relies on patient history and immune status, while predictive biomarkers remain an essential avenue for future investigation [107,108,109].

### 4.2. Managing Vaccine-Associated Complications

High fever, hepatic dysfunction, renal failure, hypercoagulability, and platelet dysfunction are manifestations of yellow fever (YF), an exceedingly rare but serious adverse event (SAE) after vaccination. An infrequent adverse effect, termed YEL-AND, manifested after a YF vaccination. When 53 case reports were examined, 38 were found to have meningoencephalitis, 7 to have Guillain-Barré Syndrome, 6 to have Acute Disseminated Encephalomyelitis, and 5 to have myelitis. Patients diagnosed with YEL-AND who also had GBS, ADEM, or myelitis had a terrible prognosis [110]. Five occurrences of encephalitis and one case of acute disseminated encephalomyelitis were identified as being caused by the attenuated vaccination (YEL), according to the study’s examination of neurologic adverse effects following immunization. YEL rarely causes encephalitis post-vaccination, and recovery is common in healthy adults. The relationship between ADEM and GBS is uncertain. YEL is indicated for wild-type YFV exposure, low reporting rates, and no fatalities [111]. In high-risk regions, the YF vaccine is recommended because its benefits outweigh its risks and are safe and effective. There have been reports of significant adverse effects, even though YFV infection is more fatal than SAEs. Early detection and management, communication of immunization benefits and hazards, and prevention of misinformation are all responsibilities of healthcare staff [112].

### 4.3. Balancing Risk and Benefit: The Yellow Fever Vaccination Dilemma

The severity of yellow fever and the proven effectiveness of its vaccine present a challenge in balancing disease prevention with the risk of adverse reactions, particularly among individuals predisposed to vaccine-related complications. Until a safer alternative to the 17D vaccine is developed, clinicians must carefully weigh the benefits and risks when advising travelers. For endemic regions, policymakers should prioritize both vaccination coverage and safety. Although the vaccine carries a far lower risk of death than infection with the wild-type virus, the factors predisposing certain immunologically sensitive travelers to severe adverse events remain unclear. Most yellow fever cases occur in tropical America, where migrant workers can facilitate disease spread as they move through forested interiors and edge habitats [113].

Between 1999 and 2009, Brazil reported 392 cases, 90% of which occurred among unvaccinated individuals. The vaccine was not often administered in some areas of southern Brazil during an outbreak in 2008 and 2009 that caused substantial mortality in monkeys and humans. The study examined the hazards and benefits of the YF vaccine at this time [114]. Brazil’s endemic yellow fever (YF) vaccine, the 17DD, has been linked to four fatal adverse events, highlighting the need for adequate vaccine coverage and surveillance data. The study suggests cautious prevention, with a risk of 1 death per million doses [115].

Older individuals are at increased risk of developing serious adverse events such as yellow fever vaccine–associated viscerotropic disease (YEL-AVD) and neurotropic disease (YEL-AND). This heightened susceptibility is thought to be multifactorial. Age-related immune decline, or immunosenescence, plays a central role: in older adults, both innate and adaptive immune responses are less efficient, leading to impaired viral clearance and altered regulation of immune activation. This diminished control may allow the attenuated 17D vaccine virus to replicate unchecked in peripheral tissues or spread to the central nervous system [116,117,118].

Comorbidities that are more common in older individuals—such as cardiovascular disease, diabetes, or chronic inflammatory conditions—further contribute to the risk by disrupting immune homeostasis and increasing systemic vulnerability. Polypharmacy and underlying subclinical organ dysfunction (e.g., hepatic or renal impairment) may also exacerbate disease severity by altering vaccine virus metabolism and host defense mechanisms. Thus, the increased incidence of viscerotropic and neurotropic disease in this age group reflects both biological immune aging and the cumulative burden of comorbidities, which together impair the host’s ability to balance protective immunity with safe containment of the vaccine virus [119,120,121].

## 5. Yellow Fever Outbreaks and Control Measures

The mosquito-borne virus known as yellow fever can cause devastating epidemics in the tropical regions of South America and Africa. The rapid spread of the YF virus via aircraft is a significant public health concern, as mosquitoes primarily transmit the infection. Climate, ecology, socioeconomic status, and politics are among the factors that influence the emergence of the virus. Through rapid reaction and urban development, the World Health Organization seeks to safeguard populations, stop the global spread of diseases, and limit epidemics [122]. Yellow fever outbreaks threaten large populations in South America and Africa, affecting local mosquito populations, virus strain, and sociopolitical factors. The WHO EYE plan aims to control YF from 2017 to 2026, but limited vaccine supplies and increasing global travel pose risks. Resources, political will, and leadership are needed to control YF [123].

International air travel regulations help prevent the spread of yellow fever, primarily through the International Health Regulations (IHR) (https://apps.who.int/gb/ebwha/pdf_files/WHA58/WHA58_3-en.pdf, accessed on 1 December 2025). Travelers from endemic regions must present a valid International Certificate of Vaccination or Prophylaxis (ICVP) to demonstrate vaccination at least 10 days before travel, thereby reducing the risk of introducing the virus into new areas. In parallel, aircraft disinsection is mandated in many countries to eliminate mosquitoes that could carry the virus, and airports in endemic zones often enforce vector surveillance. Together, vaccination requirements and vector control provide a dual safeguard against transmission via air travel (https://www.who.int/health-topics/international-health-regulations, accessed on 1 December 2025).

### 5.1. Recent Yellow Fever Outbreaks: Lessons Learned and Challenges Faced

The yellow fever virus, which causes yellow fever, is a significant public health concern worldwide. In 2016, Brazil experienced a significant epidemic, causing widespread deaths in previously unaffected areas. The epidemic increased significantly from 2016 to 2019, causing economic burdens on health authorities and public health [124]. Yellow fever epidemics have all had a severe impact on the healthcare system, people, and the economy. Eliminating worldwide outbreaks, particularly in African countries like Nigeria, requires the EYE strategy. Intermittent outbreaks occurred in Nigeria from 2017 to 2019, but better preparedness, faster responses, and more accurate reporting are required to reduce illness and death [125]. In regions such as Africa and South America, where resources are scarce, diseases like yellow fever persist despite the availability of an effective vaccine. A pandemic in eastern Senegal was monitored by the Senegalese Ministry of Health, the WHO, the 4S network, and the Institut Pasteur de Dakar in 2020 and 2021 [126]. Urban epidemics of yellow fever are expected to occur in South America, where the disease is currently spreading. Sylvatic cycles, vector control initiatives, immunization, monitoring, and case management have not been effective in preventing or controlling recent epidemics. Although urban *Aedes*-human YF outbreaks are unlikely to occur, they nevertheless necessitate well-planned public health interventions [127].

During the 2020–2021 yellow fever outbreak in eastern Senegal, national and international partners—including the Ministry of Health, WHO, Pasteur Institute of Dakar, and the 4S surveillance network—coordinated an effective response that enabled rapid case detection and control. A notable innovation was the use of genomic surveillance, which identified the circulating strain, linked it to a West African lineage, and detected a unique viral-genome deletion, thereby enhancing epidemiological insights. While genomic tools were central, there is no evidence that AI-based prediction or mobile laboratories were employed during this outbreak [126].

### 5.2. Role of Vaccination in Controlling Epidemics

Yellow fever vaccines control global epidemics by eliciting immune responses that recognize and neutralize pathogens, providing individual protection and contributing to herd immunity, thereby halting disease transmission [128]. In this study, controlling infection is crucial, as Angola experienced an emerging yellow fever outbreak, with cases reported in other African countries and China (Table 4). A greater number of Chinese workers in Angola may not have had access to the yellow fever vaccine, raising concerns about the potential spread of the disease in Asia. The area is likely to experience dengue fever outbreaks due to the abundance of *Aedes aegypti* mosquitoes, the primary vectors of the disease.

Our emergency supply of vaccines is dwindling, and our monitoring systems are still relatively new. This is a significant threat to global health [139]. Researchers developed a spatiotemporal model of yellow fever to predict the likelihood of outbreaks worldwide and the number of vaccinations required to eradicate them. The study indicated that risk varies substantially, with the Equatorial region of Latin America and West Africa as the primary regions of high-risk transmission. The study found that, to eradicate YF outbreaks worldwide, a higher level of population immunity is required than the WHO recommends (80%), and that each endemic country should develop its vaccination plan based on its risk profile [140]. Based on the provided data, two types of ecological niche models were created to assess the severity of yellow fever in sub-Saharan Africa. These models were used to predict future mass vaccination programs, vaccine demand, and illness and mortality rates across regions. In light of this data, international efforts to combat the rising threat of yellow fever can be better planned [141]. The study reveals a decrease in yellow fever vaccine doses in Brazil before and during the pandemic, with stationary behavior in five regions, but an increasing trend in Alagoas State and Roraima State [142].

The use of fractionated doses of the yellow fever vaccine has emerged as a critical strategy to address vaccine shortages during epidemics. Administering one-fifth of the standard dose intramuscularly has demonstrated sufficient immunogenicity to confer short-term protection, as evidenced during the 2016 Angola outbreak and later campaigns in Brazil and the Democratic Republic of the Congo [143,144,145,146]. The WHO currently supports this approach for emergency use, especially when full-dose supplies are constrained. However, challenges remain: the duration of protection from fractionated doses is not fully established, especially in children and immunocompromised populations [143,144,145,146,147]. Additionally, this approach is not yet licensed for routine immunization or travel-related prophylaxis. Despite these limitations, fractionated dosing remains a valuable tool for rapid mass immunization, reducing transmission during urban outbreaks and enhancing outbreak preparedness. Its continued assessment and potential formal integration into immunization strategies require further clinical studies and regulatory consensus [143,144,145,146,148].

### 5.3. Integrating Vaccination Strategies with Vector Control

A comprehensive approach that combines vaccination and vector control measures is essential for controlling vector-borne diseases, such as yellow fever, dengue, malaria, and Zika, targeting both human and environmental factors [149]. YFV is transmitted through three cycles: sylvatic, intermediate, and urban, with the sylvatic cycle occurring between rainforest primates, the intermediate cycle in isolated rural communities, and the urban cycle facilitated by invasive, domesticated mosquitoes [150]. Following a period of low vaccination coverage, the vector-borne disease yellow fever has resurged in tropical Africa and South America. To curb future epidemics, recent mass vaccination initiatives have resulted in a 27% reduction in both cases and deaths [151]. A deterministic model for vector-borne diseases with vaccines identifies mosquito fertility reduction as the most effective measure, with the highest vaccination rate resulting in the least mortality [152].

## 6. Future Directions in Yellow Fever Vaccination

Yellow fever vaccination strategies must evolve to address global health changes, focusing on improving efficacy, expanding access, and integrating innovative technologies for stronger disease control. The need for new YF vaccine candidates is increasing due to limited seed lot systems, limited manufacturing capabilities, climate change, and the rapid spread of epidemics. Current YF vaccine candidates include inactivated, recombinant, plasmid-vectored, virus-like particle, mRNA, and plant-produced subunit vaccines, utilizing the 17D genetic backbone [153].

The transition from preclinical success to human trials for mRNA yellow fever vaccines is constrained by several barriers: the availability of a highly effective, licensed vaccine (YF-17D) complicates trial justification; safety and durability remain untested in humans; and manufacturing and cold-chain stability pose challenges. Additionally, limited market demand in endemic regions reduces investment and slows clinical advancement [153].

The Brighton Collaboration V3SWG notes key risks associated with recombinant viral vectors carrying heterologous genes: potential recombination with wild-type viruses, biodistribution to unintended tissues, and immune-related concerns, including reactogenicity, autoimmunity, and reduced efficacy due to pre-existing vector immunity. Additional issues include shedding/transmission and rare risks of insertional mutagenesis or oncogenesis, underscoring the need for rigorous preclinical testing and harmonized safety monitoring [154].

One of the remarkable features of the yellow fever 17D vaccine is its continued effectiveness despite the incomplete understanding of its attenuation mechanism. Unlike many modern vaccines developed through rational design, 17D was generated empirically in the 1930s by serial passage of wild-type virus in chicken embryos, resulting in reduced pathogenicity while preserving immunogenicity. The precise molecular basis of attenuation remains elusive, as multiple mutations across structural and nonstructural genes likely contribute in a synergistic manner rather than through a single defined locus [155,156,157]. Nevertheless, the vaccine consistently induces strong and durable immune responses, with robust neutralizing antibodies against the envelope protein and potent CD8^+^ T-cell memory, which together provide lifelong protection in most recipients. Significantly, attenuation has not compromised the presentation of critical antigenic epitopes, ensuring that immune responses closely mimic those elicited by wild-type infection [66,155,158]. This combination of broad humoral and cellular immunity explains why 17D has maintained exceptional efficacy for nearly a century, even in the absence of a fully elucidated attenuation mechanism. Ongoing advances in molecular virology, reverse genetics, and systems immunology may ultimately clarify the determinants of attenuation, but the empirical success of 17D highlights that robust protective immunity can be achieved independently of a complete mechanistic understanding [51,157,159].

### 6.1. Advancements in Vaccine Technology: Novel Approaches and Platforms

Advancements in vaccine technology have significantly improved safety, efficacy, speed, and adaptability, paving the way to address future global health challenges, such as pandemics and infectious diseases for which effective vaccines are lacking. Researchers in the UK found that mRNA vaccine candidates protected mice and rhesus macaques from the lethal YF virus by eliciting protective immune responses. Based on these results, these mRNA vaccines may be a suitable option for the permitted YF vaccine supply, which could aid in future epidemic prevention efforts by reducing vaccine shortages [160]. The V3SWG, which stands for the Brighton Collaboration Viral Vector vaccines Safety Working Group, conducts risk assessments of live, recombinant viral vaccines that contain heterologous viral genes. An authorized vaccine against Japanese encephalitis was one of several that used genes from dengue and West Nile viruses [161]. Engineered nanoparticles show potential as vaccine delivery platforms, enhancing mucosal and systemic immunity. However, clinical translation and the development of broad-spectrum vaccines are crucial due to cost and antigenic drift [162].

Future research and development in yellow fever vaccine technology should focus on several critical areas. First, innovative vaccine platforms such as mRNA-based or viral-vectored approaches could provide safer alternatives for populations with contraindications to the current live-attenuated vaccine. Second, efforts are needed to develop thermostable formulations that do not require a cold chain, thereby significantly enhancing accessibility in remote and resource-limited settings [163,164]. Third, fractional dosing strategies should continue to be evaluated for long-term immunogenicity, especially in outbreak situations with limited vaccine supply. Finally, ongoing research must address rare but serious adverse events by improving understanding of host–pathogen–vaccine interactions, thereby ensuring safety without compromising efficacy. These directions will be vital for strengthening yellow fever prevention in a changing global landscape [165,166].

### 6.2. Next-Generation Vaccine Platforms for Yellow Fever

The 17D live-attenuated vaccine remains the cornerstone of yellow fever prevention due to its excellent single-dose efficacy and long-term durability. Nevertheless, it has recognized limitations, including contraindications in immunocompromised individuals, older adults, and pregnant women, as well as reliance on egg-based production methods that can constrain supply. These factors have stimulated interest in next-generation vaccine platforms, particularly mRNA and non-replicating viral vector approaches, which may complement the 17D vaccine by addressing both safety and manufacturing challenges [69,167,168].

mRNA vaccines offer several advantages, most notably their safety profile, as they are noninfectious and pose no risk of viscerotropic or neurotropic disease. By encoding the prM–E antigens, they can generate subviral particles that display the quaternary epitopes necessary for potent neutralizing antibody responses. Their production is egg-independent, scalable, and adaptable, making them attractive for rapid deployment during outbreaks. However, uncertainties remain regarding the durability of immune responses, as protection may require two doses or booster schedules to achieve levels comparable to those achieved with 17D. Cold-chain requirements, although improving with newer lyophilized and thermostable formulations, also present challenges in tropical endemic regions [169,170,171].

Viral vector platforms, such as adenovirus-based or measles-vectored vaccines, likewise provide strong immunogenicity, often inducing both neutralizing antibodies and robust CD4^+^ and CD8^+^ T-cell responses. These vectors are non-replicating and thus avoid the risks associated with live attenuated yellow fever virus. Many have favorable stability profiles and can be manufactured at large scale in egg-free systems. Potential limitations include pre-existing vector immunity, which could reduce vaccine effectiveness, and regulatory challenges, as clinical evaluation would require immunobridging studies against the well-established 17D vaccine. Vector-specific adverse events, though rare, would also need careful monitoring [24,165,172].

From a programmatic standpoint, both mRNA and viral vector vaccines could expand global supply capacity and reduce reliance on egg-based production, a critical need during outbreak response and mass immunization campaigns. They may be handy for populations unable to receive 17D safely, as well as for heterologous prime-boost strategies designed to enhance the breadth and durability of immunity. Importantly, their role in fractional dosing and dose-sparing approaches warrants exploration, as this could extend vaccine stockpiles further [173,174,175].

Immunologically, the critical correlate of protection remains the induction of neutralizing antibodies against the envelope (E) protein, and platforms that successfully present native-like E protein epitopes are the most promising. Viral vectors typically elicit stronger cellular responses, whereas newer approaches, such as self-amplifying RNA, may enhance T-cell immunity at lower doses. Operational aspects, including thermostability, last-mile delivery, and cost-effectiveness, will be decisive in determining their applicability in endemic and resource-limited settings [155,158,176].

In summary, mRNA and viral vector-based vaccines hold the potential to provide safer alternatives for populations contraindicated for 17D and to enhance global manufacturing resilience. While they may not yet surpass 17D in long-term protection, they could play a valuable complementary role in outbreak preparedness, stockpile expansion, and immunization of high-risk groups. Over time, with supportive durability and field-effectiveness data, these technologies may emerge as programmatic alternatives that further strengthen yellow fever prevention strategies [24,118,177].

### 6.3. Targeting Vulnerable Populations: Vaccination Equity and Accessibility

To control diseases such as yellow fever, it is essential to achieve equitable vaccination coverage. However, there are hurdles to vaccine access for vulnerable people; thus, a holistic approach is needed. The research employs environmental factors to assess the susceptibility of Brazilian communities to a high burden of YF. Mild, low, and high levels of vulnerability were used for classification. Using a cumulative logit model, the North and Central-West regions were identified as the most vulnerable. Prioritizing YF surveillance and prevention is made easier by the results [178]. The EYE strategy targets 40 high-risk countries to increase yellow fever vaccine coverage. However, coverage rates vary widely, with significant differences across the United States and Africa. This may be due to differences in target populations and vaccine availability [179]. The WHO is employing dose-sparing strategies for its immunization initiative in Kinshasa, the Democratic Republic of the Congo, in response to the yellow fever epidemic in Angola. The 5-fold fractional-dose vaccination, evaluated for efficacy but not yet tested, can diminish infectious attack rates if efficacy surpasses 20% [180].

### 6.4. Strengthening Surveillance and Monitoring for Vaccine-Preventable Diseases

Surveillance systems are crucial for preventing, controlling, and eradicating vaccine-preventable diseases, particularly in a globalized world where outbreaks can spread rapidly. Between 1999 and 2001, 67 confirmed cases and 42% of vaccinations occurred in Rio Grande do Sul, Brazil, primarily due to the use of the yellow fever vaccine in monkey surveillance. The distribution of vaccines and the prevention of YF in susceptible human populations are both greatly assisted by epizootic surveillance [181]. Brazil experienced a 33.6% yellow fever case fatality rate, primarily in southern states with low vaccination coverage, highlighting the need for the World Health Organisation’s “Global Strategy to Eliminate Epidemic” [182]. A surveillance network in Minas Gerais, Brazil, confirmed the first YFV case in non-human primates in 2021, highlighting the need for coordinated surveillance and contingency measures to prevent YFV spillover to humans [183]. The objective of the 2007 GFIMS was to consolidate surveillance systems to enhance national monitoring of vaccine-preventable illnesses (VPDs). Conversely, the establishment of integrated VPD monitoring has proven to be a formidable undertaking [184].

An illustrative example of the critical role of early detection and rapid response is the 2021 outbreak in Minas Gerais, Brazil. Following the sudden die-off of non-human primates, a coordinated surveillance system integrating digital tools (SISS-Geo), rapid molecular diagnostics (RT-qPCR), and real-time genomic sequencing (Nanopore) confirmed the presence of a new South American genotype I sub-lineage (YFV PA/MG). This swift confirmation enabled the immediate launch of targeted vaccination campaigns, effectively preventing human spillover and underscoring the importance of timely interventions before broader epidemic spread [183].

## 7. Yellow Fever in the Context of Emerging Infectious Diseases

Yellow fever, a mosquito-borne disease, poses a significant public health concern due to its sporadic outbreaks and rapid spread, particularly in urban areas. Despite the effectiveness of vaccines, the risks of emerging infectious diseases remain. Yellow fever, a feared infectious disease, is re-emerging due to increased human migration and low vaccination rates, necessitating stricter regulation, border checks, alternative vaccine research, and global efforts [185].

### 7.1. Yellow Fever as a Model for Preparedness and Response

Yellow fever offers valuable lessons for public health systems in disease surveillance, vaccination strategies, and global collaboration, thereby enhancing response capabilities and preventing large-scale outbreaks, making it a critical model for addressing future public health threats. Brazil’s YFV surveillance relies heavily on entomovirological monitoring, which facilitates the connection between human and non-human primate transmission. Identifying unique or prospective vectors can elucidate their role in the propagation and maintenance of sylvatic YFV. Consequently, vector control, vaccination rates, and monitoring all need to be intensified [186]. Yellow fever, a mosquito-transmitted disease, is spreading in previously unaffected areas due to enzootic cycles in the Amazon basin of South America and Brazil, as well as its westward spread in Africa [187]. A multidisciplinary study conducted in Colombia examined arbovirus transmission and found that both Aedes aegypti mosquitoes in cities and sylvatic mosquitoes near cities had high infection rates. The study found that early risk factors for transmission in rural areas include climate, socioeconomic status, and human activities [188].

Vector control in forested areas is limited compared to urban zones. Unlike *Aedes aegypti* in cities, forest vectors such as *Haemagogus* and *Sabethes* breed in inaccessible sites, such as tree holes, making interventions impractical. Insecticide use is logistically challenging and environmentally unsustainable, so vaccination remains the most effective prevention strategy in these regions [189].

### 7.2. Potential Cross-Protection with Other Flaviviruses

Flaviviruses comprise a group of medically necessary viruses that include yellow fever, dengue, Japanese encephalitis, West Nile, Murray Valley encephalitis, Saint Louis encephalitis, and Zika viruses. They belong to the genus *Flavivirus* within the family *Flaviviridae* and are classified into three categories: tick-borne, mosquito-borne, and those without known vectors [190]. Early research on cellular immunity to dengue virus (DENV) showed easily detectable T cell responses and serotype cross-reactivity in both CD4^+^ and CD8^+^ T cells. Serotype cross-reactivity is influenced by targeting immunodominant responses, with the most cross-reactive responses being directed against tetravalent DENV vaccines, including NS proteins [191]. It is common for travelers, particularly those taking VFRs, to contract typhoid and paratyphoid fever, which are worldwide health concerns. A greater emphasis on preventative actions, including immunizations, is warranted in light of the increasing prevalence of antimicrobial resistance [192]. A study highlights the need to assess the protective scope of live attenuated viruses (LAVs) against African swine fever (ASF), as their effectiveness is limited due to the diverse ASF isolates [193].

### 7.3. One Health Approach: Integrating Animal and Human Health

The yellow fever case study underscores the interconnectedness of human, animal, and environmental health, exemplifying the One Health approach. Its sylvatic transmission cycle involves diverse insect vectors and non-human primates, with mosquitoes of the genera *Haemagogus* and *Sabethes* playing a central role. Human infection can occur when infected mosquitoes bite people in or near forested areas. Surveillance of non-human primate mortality (epizootics) serves as an essential early warning system for potential human outbreaks. Brazil has effectively implemented monkey population monitoring as part of its yellow fever prevention and epidemic response strategy [194]. Deforestation, urban expansion, and climate change further complicate control efforts by altering vector habitats and increasing human exposure to sylvatic transmission cycles [195]. A coordinated One Health strategy integrates wildlife surveillance, vector control, environmental management, and human vaccination to prevent and control yellow fever outbreaks effectively. This holistic model is essential not only for yellow fever but also for addressing other emerging zoonotic threats [196].

Climate change and rapid urbanization are expected to profoundly influence the risk of yellow fever outbreaks beyond traditionally endemic areas. Rising global temperatures, changes in precipitation patterns, and altered seasonality are expanding the ecological niches of *Aedes aegypti* and *Aedes albopictus*, enabling their establishment in temperate regions previously unsuitable for sustained transmission [197,198]. Concurrently, unplanned urban growth creates densely populated environments with inadequate water, sanitation, and waste management systems, favoring mosquito breeding and amplifying transmission potential. Increased international travel and trade further heighten the risk of introducing yellow fever into non-endemic regions with immunologically naïve populations [199], where outbreaks could be explosive if vaccination coverage is low [27,37]. Together, these drivers underscore the urgency of strengthening surveillance, maintaining robust vaccination programs, and integrating climate-informed vector control into preparedness strategies for regions at emerging risk [200,201].

## 8. Limitations

This review has several limitations that should be acknowledged. First, although we aimed to provide a comprehensive and up-to-date synthesis of the literature on yellow fever vaccination, the field’s rapid evolution means that newly emerging data may not be included. Second, the review primarily draws from published literature in English, Portuguese, and Spanish, which may exclude relevant studies in other languages, especially those from endemic regions. Third, data on certain aspects—such as the long-term immunogenicity of fractionated doses and vaccine efficacy in immunocompromised populations—remain limited or inconclusive, which impacts the depth of analysis in these areas. Additionally, although this review encompasses both historical and recent findings, it does not conduct a formal systematic review or meta-analysis, which limits its ability to quantify outcomes. These limitations underscore the need for continued research and for more robust multicenter clinical studies to inform vaccination policies and outbreak preparedness strategies.

## 9. Conclusions

Yellow fever vaccine development has made significant progress, yet essential challenges remain in achieving comprehensive disease control. Addressing gaps in vaccination coverage, managing urban outbreaks, and ensuring vaccine availability during emergencies require innovative vaccine technologies, integrated strategies, and strengthened surveillance systems. The YF-17D live-attenuated vaccine, developed in 1937, revolutionized yellow fever prevention by providing long-lasting protection with a single dose. Large-scale vaccination campaigns in South America and Africa have substantially reduced the disease burden. At the same time, global initiatives, such as the World Health Organization’s stockpile system, have improved preparedness for outbreaks.

Nevertheless, achieving universal vaccination remains difficult given current production constraints and fluctuating global demand. Although the WHO emergency stockpile can be mobilized relatively quickly, supply shortages and logistical hurdles may delay a timely response. Regional reserves serve as essential buffers, but their adequacy varies across high-risk areas, underscoring the urgent need to expand manufacturing capacity, secure reliable distribution networks, and maintain equitable access to vaccines.

Looking forward, novel vaccine platforms—including DNA, mRNA, and viral vector-based technologies—hold promise to enhance efficacy, safety, and scalability. The roadmap toward a yellow fever–free future must combine these advances with effective vector control, expanded laboratory capacity, and robust global surveillance networks. Achieving vaccination equity is paramount, ensuring vulnerable and underserved populations are reached through both routine immunization and emergency campaigns. While significant progress has been made, sustainable investment, scientific innovation, and coordinated action from governments, public health agencies, and international organizations remain essential to overcoming persistent challenges and ultimately eradicating yellow fever as a global health threat.

## Figures and Tables

**Table 1:** Yellow fever vaccine coverage rates (%) in Africa and the Americas, 2000–2024.

Year	Africa (%)	Americas (%)	Year	Africa (%)	Americas (%)
2000	9	24	2013	37	55
2001	10	28	2014	40	56
2002	12	18	2015	41	55
2003	15	32	2016	40	53
2004	26	34	2017	43	54
2005	33	40	2018	47	56
2006	36	52	2019	47	61
2007	39	52	2020	46	58
2008	39	51	2021	45	58
2009	43	44	2022	43	56
2010	40	45	2023	47	63
2011	35	49	2024	50	67
2012	35	54			

Source: https://immunizationdata.who.int/global/wiise-detail-page/yellow-fever-(yf)-vaccination-coverage?GROUP=WHO_REGIONS&YEAR=&CODE=, accessed on 1 December 2025.

**Table 2:** Key genetic mutations in yellow fever vaccine substrains compared to the parental strain.

Gene/Region	Mutation Type	Parental	17D	17D-204	17DD (YF-17D-213/77)	Functional/Phenotypic Relevance *
prM/E	Amino acid substitution	Residue X → Y	Change A	Change B	Change C	Altered virion maturation, attenuation
NS1	Nucleotide substitution	nt ###	Mut 1	Mut 2	Mut 3	Immune modulation
NS2A	Amino acid substitution	Residue M	Mut A	Mut B	Mut C	Replication efficiency
NS3	Substitution/deletion	Residue ##	Mut A	Mut B	Mut C	Protease/helicase activity
NS4B	Substitution	Residue ##	Mut A	Mut B	Mut C	Interferon sensitivity
NS5 (RdRp)	Substitution	Residue ##	Mut A	Mut B	Mut C	Polymerase fidelity

**Table 4:** Main recent yellow fever epidemics.

Number	Outbreak and Location	Year	Result	Reference
1.	Angola and Brazil	1970–2016	Yellow fever risk zones still have 393–472.9 million individuals who require vaccination to meet the World Health Organization’s 80% coverage target, despite substantial growth in vaccine coverage since 1970.	[18]
2.	Angola and Brazil	2015–2016	The 2016 YF outbreak in Luanda, Angola, was analyzed using a vector-host epidemic model, revealing that timely vaccination and behavioral changes can reduce deaths and prevent future outbreaks.	[129]
3.	Uganda (East Africa)	2019–2022	The proposal proposes establishing a YF elimination task force to coordinate surveillance, vaccination campaigns, mosquito management strategies, and risk communication to reduce YF incidence and outbreaks.	[130]
4.	Brazil	2016–2017	Due to the presence of animal reservoirs, human susceptibility, and vector-borne transmission, unvaccinated travelers to the affected states of Brazil are at risk of contracting the virus. A potential pandemic could be triggered by ecological conditions and enzootics, potentially leading to spillover.	[131]
5.	Brazil	2017–2018	In 2016, Brazil experienced the largest yellow fever outbreak in the Americas, primarily in densely populated areas like São Paulo, originating from three South American genotype variants.	[132]
6.	West African and South American	2001–2003	Yellow fever, a tropical ailment responsible for 200,000 cases and 30,000 fatalities each year, is spread by humans, mosquitoes, and monkeys, with the possibility of preventative and chimeric vaccines.	[133]
7.	Brazil	2016–2017	The YFV outbreak in Brazil necessitates prompt detection and control through epidemiological and genetic surveillance, supported by a global plan to eradicate epidemics by 2026.	[134]
8.	Brazil	2016–2018	UYF prevention relies on insect control measures, insecticide resistance, behavioral interventions, and health surveillance; however, recent outbreaks in Brazil have demonstrated the ineffectiveness of these measures.	[135]
9.	Angola	2015–2016	Despite multiple vaccination campaigns, the Angola YFV outbreak reached its peak in February 2016, with 4347 suspected cases and 377 deaths, leading to an emergency campaign in August 2016.	[136]
10.	Angola	2015	Yellow fever rapidly spreads from Luanda, Angola, with 49 districts reporting cases within three months. Prioritizing vaccination is recommended; however, constraints such as vaccine supply and delivery logistics must also be considered.	[137]
11.	Brazil and Venezuela	1990 to 2022	Nine patients with YF-compatible symptoms in French Guiana, Venezuela, Suriname, and Brazil died within 8 days, requiring stronger vaccination coverage due to the likely persisting sylvatic cycle.	[138]
12.	South American countries	2024–2025	Current epidemics with more than 350 cases and more than 150 deaths are associated with a lack of vaccinations in certain age groups in Colombia and Brazil, which have concentrated most of the cases.	https://shiny.paho-phe.org/yellowfever/ (accessed on 1 December 2025)

## Data Availability

Not directly applicable, as is a Review Article.

## References

[1] Angerami R.N., Socorro Souza Chaves T.D., Rodríguez-Morales A.J. (2025). Yellow fever outbreaks in South America: Current epidemiology, legacies of the recent past and perspectives for the near future. New Microbes New Infect..

[2] Srivastava S., Dhoundiyal S., Kumar S., Kaur A., Khatib M.N., Gaidhane S., Zahiruddin Q.S., Mohanty A., Henao-Martinez A.F., Krsak M. (2024). Yellow Fever: Global Impact, Epidemiology, Pathogenesis, and Integrated Prevention Approaches. Infez. Med..

[3] Reno E., Quan N.G., Franco-Paredes C., Chastain D.B., Chauhan L., Rodriguez-Morales A.J., Henao-Martínez A.F. (2020). Prevention of yellow fever in travellers: An update. Lancet Infect. Dis..

[4] Bassey B.E., Braka F., Onyibe R., Kolude O.O., Oluwadare M., Oluwabukola A., Omotunde O., Iyanda O.A., Tella A.A., Olanike O.S. (2022). Changing epidemiology of yellow fever virus in Oyo State, Nigeria. BMC Public Health.

[5] Kallas E.G., D’Elia Zanella L., Moreira C.H.V., Buccheri R., Diniz G.B.F., Castiñeiras A.C.P., Costa P.R., Dias J.Z.C., Marmorato M.P., Song A.T.W. (2019). Predictors of mortality in patients with yellow fever: An observational cohort study. Lancet Infect. Dis..

[6] Blake J.B. (1968). Yellow fever in eighteenth century America. Bull. N. Y. Acad. Med..

[7] Barrett A.D., Higgs S. (2007). Yellow fever: A disease that has yet to be conquered. Annu. Rev. Entomol..

[8] Alshahrani N.Z., Algethami M.R., Albeshry A.M., Awan Z., Alzhrani W., Fairaq B.A., Rashid H. (2025). Respiratory symptom burden, vaccination coverage, and preventive health practices among Sudanese Hajj pilgrims who traveled by sea. Front. Public Health.

[9] Thomas R.E. (2016). Yellow fever vaccine-associated viscerotropic disease: Current perspectives. Drug Des. Dev. Ther..

[10] Petersen J.L. (1977). Behavioral Differences in Two Subspecies of *Aedes aegypti* (L.) (Diptera: Culicidae) in East Africa. Ph.D. Thesis.

[11] Cuéllar-Sáenz J.A., Rodríguez-Morales A.J., Faccini-Martínez Á.A. (2025). Reemergence of Yellow Fever, Magdalena Valley, Colombia, 2024–2025. Emerg. Infect. Dis..

[12] Sanchez-Rojas I.C., Bonilla-Aldana D.K., Solarte-Jimenez C.L., Bonilla-Aldana J.L., Belisario-Tovar M., Ortega-Gómez S., Zambrano-Quenan V.M., Perafan-Gomez J.C., Gomez-Ocampo C.H., Delgado-Cajigas M. (2025). Fatal yellow fever among captive non-human primates in southern Colombia, 2025. Front. Vet. Sci..

[13] Bonilla-Aldana D.K., Bonilla-Aldana J.L., Castellanos J.E., Rodriguez-Morales A.J. (2025). Importance of Epizootic Surveillance in the Epidemiology of Yellow Fever in South America. Curr. Trop. Med. Rep..

[14] Escalera-Antezana J.P., Aviles-Sarmiento J.L., Montenegro-Narvaez C.M., Castro-Calderon H.A., Bonilla-Aldana J.L., Bonilla-Aldana D.K., Rodriguez-Morales A.J. (2025). Yellow Fever among Captive Non-Human Primates in La Paz, Bolivia, 2025. New Microbes New Infect..

[15] Sanchez-Rojas I.C., Solarte-Jimenez C.L., Chamorro-Velazco E.C., Diaz-Llerena G.E., Arevalo C.D., Cuasquer-Posos O.L., Bonilla-Aldana J.L., Bonilla-Aldana D.K., Rodriguez-Morales A.J. (2025). Yellow fever in Putumayo, Colombia, 2024. New Microbes New Infect..

[16] Tuboi S.H., Costa Z.G.A., da Costa Vasconcelos P.F., Hatch D. (2007). Clinical and epidemiological characteristics of yellow fever in Brazil: Analysis of reported cases 1998–2002. Trans. R. Soc. Trop. Med. Hyg..

[17] Monath T.P., Nichols R., Archambault W.T., Moore L., Marchesani R., Tian J., Shope R.E., Thomas N., Schrader R., Furby D. (2002). Comparative safety and immunogenicity of two yellow fever 17D vaccines (ARILVAX and YF-VAX) in a phase III multicenter, double-blind clinical trial. Am. J. Trop. Med. Hyg..

[18] Shearer F.M., Moyes C.L., Pigott D.M., Brady O.J., Marinho F., Deshpande A., Longbottom J., Browne A.J., Kraemer M.U., O’Reilly K.M. (2017). Global yellow fever vaccination coverage from 1970 to 2016: An adjusted retrospective analysis. Lancet Infect. Dis..

[19] Frierson J.G. (2010). The yellow fever vaccine: A history. Yale J. Biol. Med..

[20] Lindsey N.P., Schroeder B.A., Miller E.R., Braun M.M., Hinckley A.F., Marano N., Slade B.A., Barnett E.D., Brunette G.W., Horan K. (2008). Adverse event reports following yellow fever vaccination. Vaccine.

[21] Forero-Delgadillo A.J., Morales-Olivera J.A., Celis-Guzmán J.F., Zapata-Díaz O.E., González-Varona G.A., Acevedo-Bedoya C.A., Salazar-Fernández R., Ordoñez J.O., Robayo-Amortegui H., Quintero-Altare A. (2025). Colombian consensus on the care of critically ill patients with suspected or confirmed severe yellow fever. Lancet Reg. Health Am..

[22] Monath T.P. (2007). Dengue and yellow fever—Challenges for the development and use of vaccines. N. Engl. J. Med..

[23] Barrett A.D., Teuwen D.E. (2009). Yellow fever vaccine—How does it work and why do rare cases of serious adverse events take place?. Curr. Opin. Immunol..

[24] Bassi M.R., Larsen M.A., Kongsgaard M., Rasmussen M., Buus S., Stryhn A., Thomsen A.R., Christensen J.P. (2016). Vaccination with Replication Deficient Adenovectors Encoding YF-17D Antigens Induces Long-Lasting Protection from Severe Yellow Fever Virus Infection in Mice. PLoS Negl. Trop. Dis..

[25] Sandberg J.T., Ols S., Löfling M., Varnaitė R., Lindgren G., Nilsson O., Rombo L., Kalén M., Loré K., Blom K. (2021). Activation and Kinetics of Circulating T Follicular Helper Cells, Specific Plasmablast Response, and Development of Neutralizing Antibodies following Yellow Fever Virus Vaccination. J. Immunol..

[26] Wieten R.W., Jonker E.F., van Leeuwen E.M., Remmerswaal E.B., Ten Berge I.J., de Visser A.W., van Genderen P.J., Goorhuis A., Visser L.G., Grobusch M.P. (2016). A Single 17D Yellow Fever Vaccination Provides Lifelong Immunity; Characterization of Yellow-Fever-Specific Neutralizing Antibody and T-Cell Responses after Vaccination. PLoS ONE.

[27] Rodriguez-Morales A.J., Alhazmi A.H., Katime A., Hameed A.A., Morales A., Lepetic A.C., Risquez A., Forero-Delgadillo A.J., Holguin A., Faccini-Martínez Á.A. (2025). Yellow fever in South America—A plea for action and call for prevention also in travelers from SLAMVI, ESGITM, EVASG, ALEIMC, GEPI-SEIMC, SEMEVI, and CMTZMV-ACIN. Travel. Med. Infect. Dis..

[28] Iversen E.F., Rahimic A.H.F., Frattari G.S., Rosás-Umbert M., Schleimann M.H., Olesen R., Gunst J.D., Søgaard O.S., Krogsgaard M., Tolstrup M. (2025). TCR bias drives development of dominant vaccine-induced CD8+ T cell responses which can be redirected toward cellular targets. Vaccine.

[29] Strother A.E., Thompson J.K., Widen S.G., Barrett A.D.T. (2022). Genetic Diversity Does Not Contribute to Attenuation of HeLa Passaged Wild-Type Yellow Fever Virus Strain French Viscerotropic Virus. Viruses.

[30] Pugachev K.V., Ocran S.W., Guirakhoo F., Furby D., Monath T.P. (2002). Heterogeneous nature of the genome of the ARILVAX yellow fever 17D vaccine revealed by consensus sequencing. Vaccine.

[31] Saito K., Shimasaki K., Fukasawa M., Suzuki R., Okemoto-Nakamura Y., Katoh K., Takasaki T., Hanada K. (2022). Establishment of Vero cell lines persistently harboring a yellow fever virus 17D subgenomic replicon. Virus Res..

[32] Pato T.P., Souza M.C.O., Silva A.N., Pereira R.C., Silva M.V., Caride E., Gaspar L.P., Freire M.S., Castilho L.R. (2014). Development of a membrane adsorber based capture step for the purification of yellow fever virus. Vaccine.

[33] Hong Q., Liu J., Wei Y., Wei X. (2023). Application of Baculovirus Expression Vector System (BEVS) in Vaccine Development. Vaccines.

[34] Cox M.M. (2012). Recombinant protein vaccines produced in insect cells. Vaccine.

[35] Ulmer J.B., Valley U., Rappuoli R. (2006). Vaccine manufacturing: Challenges and solutions. Nat. Biotechnol..

[36] Wiysonge C.S., Ndwandwe D., Iwu-Jaja C., Nnaji C.A., Machingaidze S., Adamu A.A., Bita Fouda A.A., Hussey G.D. (2025). Strategic positioning of immunization at the heart of Africa’s health and development agenda. Hum. Vaccines Immunother..

[37] Rodriguez-Morales A.J., Sah R., Silva-Ramos C.R., Pava-Garzón D.M. (2025). Challenges in Emerging and Reemerging Arboviral Diseases: The Examples of Oropouche and Yellow Fever. Pathogens.

[38] Okwo-Bele J.-M., Cherian T. (2011). The expanded programme on immunization: A lasting legacy of smallpox eradication. Vaccine.

[39] Cetron M.S., Marfin A.A., Julian K.G., Gubler D.J., Sharp D.J., Barwick R.S., Weld L.H., Chen R., Clover R.D., Deseda-Tous J. (2002). Yellow fever vaccine recommendations of the Advisory Committee on Immunization Practices (ACIP), 2002. Morb. Mortal. Wkly. Rep. Recomm. Rep..

[40] Mokaya J., Kimathi D., Lambe T., Warimwe G.M. (2021). What Constitutes Protective Immunity Following Yellow Fever Vaccination?. Vaccines.

[41] Gotuzzo E., Yactayo S., Cordova E. (2013). Efficacy and duration of immunity after yellow fever vaccination: Systematic review on the need for a booster every 10 years. Am. J. Trop. Med. Hyg..

[42] Mishra N., Boudewijns R., Schmid M.A., Marques R.E., Sharma S., Neyts J., Dallmeier K. (2020). A Chimeric Japanese Encephalitis Vaccine Protects against Lethal Yellow Fever Virus Infection without Inducing Neutralizing Antibodies. mBio.

[43] de Melo M.I.A., Miranda A.N.D., de Andrade A.S.R. (2025). Targeting Yellow-Fever Virus: Development of a specific aptamer to NS1 protein. J. Virol. Methods.

[44] Mateus J., Grifoni A., Voic H., Angelo M.A., Phillips E., Mallal S., Sidney J., Sette A., Weiskopf D. (2020). Identification of Novel Yellow Fever Class II Epitopes in YF-17D Vaccinees. Viruses.

[45] Lim H.X., Lim J., Poh C.L. (2021). Identification and selection of immunodominant B and T cell epitopes for dengue multi-epitope-based vaccine. Med. Microbiol. Immunol..

[46] da Silva O.L.T., da Silva M.K., Rodrigues-Neto J.F., Santos Lima J.P.M., Manzoni V., Akash S., Fulco U.L., Bourhia M., Dawoud T.M., Nafidi H.A. (2024). Advancing molecular modeling and reverse vaccinology in broad-spectrum yellow fever virus vaccine development. Sci. Rep..

[47] Reynisson B., Alvarez B., Paul S., Peters B., Nielsen M. (2020). NetMHCpan-4.1 and NetMHCIIpan-4.0: Improved predictions of MHC antigen presentation by concurrent motif deconvolution and integration of MS MHC eluted ligand data. Nucleic Acids Res..

[48] Roy R.R., Tadkalkar N., Deshpande G.R., Atre N.M., Shil P., Sapkal G. (2025). Identification of B-cell epitopes of Indian Zika virus strains using immunoinformatics. Front. Immunol..

[49] Iyyanar S., Ravi S.N. (2025). Vaccine Development T-cell (MHC-I) Epitopes Identification Against the Indian HCV Genotype: An Approach Based on Immunoinformatic. Mol. Biotechnol..

[50] Doyle M.P., Genualdi J.R., Bailey A.L., Kose N., Gainza C., Rodriguez J., Reeder K.M., Nelson C.A., Jethva P.N., Sutton R.E. (2022). Isolation of a Potently Neutralizing and Protective Human Monoclonal Antibody Targeting Yellow Fever Virus. mBio.

[51] Lou Y.N., Sun M.X., Li K., Xiong X.C., Zhou C., Cao T.S., Li X.F., Qin C.F. (2025). A single residue in domain II of envelope protein of yellow fever virus is critical for neutralization sensitivity. J. Virol..

[52] Xu Z., Kulp D.W. (2019). Protein engineering and particulate display of B-cell epitopes to facilitate development of novel vaccines. Curr. Opin. Immunol..

[53] Olotu F.A., Soliman M.E.S. (2021). Immunoinformatics prediction of potential B-cell and T-cell epitopes as effective vaccine candidates for eliciting immunogenic responses against Epstein-Barr virus. Biomed. J..

[54] Schmidt J., Smith A.R., Magnin M., Racle J., Devlin J.R., Bobisse S., Cesbron J., Bonnet V., Carmona S.J., Huber F. (2021). Prediction of neo-epitope immunogenicity reveals TCR recognition determinants and provides insight into immunoediting. Cell Rep. Med..

[55] Khan N.T., Zinnia M.A., Islam A. (2023). Modeling mRNA-based vaccine YFV.E1988 against yellow fever virus E-protein using immuno-informatics and reverse vaccinology approach. J. Biomol. Struct. Dyn..

[56] Ul-Rahman A., Shabbir M.A.B. (2020). In silico analysis for development of epitopes-based peptide vaccine against Alkhurma hemorrhagic fever virus. J. Biomol. Struct. Dyn..

[57] Silva M.L., Martins M.A., Espírito-Santo L.R., Campi-Azevedo A.C., Silveira-Lemos D., Ribeiro J.G.L., Homma A., Kroon E.G., Teixeira-Carvalho A., Elói-Santos S.M. (2011). Characterization of main cytokine sources from the innate and adaptive immune responses following primary 17DD yellow fever vaccination in adults. Vaccine.

[58] Campi-Azevedo A.C., de Araujo-Porto L.P., Luiza-Silva M., Batista M.A., Martins M.A., Sathler-Avelar R., da Silveira-Lemos D., Camacho L.A., de Menezes Martins R., de Lourdes de Sousa Maia M. (2012). 17DD and 17D-213/77 yellow fever substrains trigger a balanced cytokine profile in primary vaccinated children. PLoS ONE.

[59] Kohler S., Bethke N., Böthe M., Sommerick S., Frentsch M., Romagnani C., Niedrig M., Thiel A. (2012). The early cellular signatures of protective immunity induced by live viral vaccination. Eur. J. Immunol..

[60] Luiza-Silva M., Campi-Azevedo A.C., Batista M.A., Martins M.A., Avelar R.S., da Silveira Lemos D., Bastos Camacho L.A., de Menezes Martins R., de Lourdes de Sousa Maia M., Guedes Farias R.H. (2011). Cytokine signatures of innate and adaptive immunity in 17DD yellow fever vaccinated children and its association with the level of neutralizing antibody. J. Infect. Dis..

[61] Ferreira C.C., Campi-Azevedo A.C., Peruhype-Magalhāes V., Costa-Pereira C., Albuquerque C.P., Muniz L.F., Yokoy de Souza T., Oliveira A.C.V., Martins-Filho O.A., da Mota L.M.H. (2018). The 17D-204 and 17DD yellow fever vaccines: An overview of major similarities and subtle differences. Expert. Rev. Vaccines.

[62] Hou J., Wang S., Jia M., Li D., Liu Y., Li Z., Zhu H., Xu H., Sun M., Lu L. (2017). A Systems Vaccinology Approach Reveals Temporal Transcriptomic Changes of Immune Responses to the Yellow Fever 17D Vaccine. J. Immunol..

[63] Reis L.R., da Costa-Rocha I.A., Campi-Azevedo A.C., Peruhype-Magalhães V., Coelho-dos-Reis J.G., Costa-Pereira C., Otta D.A., Freire L.C., de Lima S.M.B., de Souza Azevedo A. (2022). Exploratory study of humoral and cellular immunity to 17DD yellow fever vaccination in children and adults residents of areas without circulation of yellow fever virus. Vaccine.

[64] Ferreira C.C., Campi-Azevedo A.C., Peruhype-Magalhāes V., Coelho-Dos-Reis J.G., Antonelli L.R.D.V., Torres K., Freire L.C., da Costa-Rocha I.A., Oliveira A.C.V., Maia M.L.S. (2019). Collaborative Group for Studies of Yellow Fever Vaccine Impact of synthetic and biological immunomodulatory therapy on the duration of 17DD yellow fever vaccine-induced immunity in rheumatoid arthritis. Arthritis Res. Ther..

[65] Hepburn M.J., Kortepeter M.G., Pittman P.R., Boudreau E.F., Mangiafico J.A., Buck P.A., Norris S.L., Anderson E.L. (2006). Neutralizing antibody response to booster vaccination with the 17d yellow fever vaccine. Vaccine.

[66] Wieten R.W., Goorhuis A., Jonker E.F.F., de Bree G.J., de Visser A.W., van Genderen P.J.J., Remmerswaal E.B.M., Ten Berge I.J.M., Visser L.G., Grobusch M.P. (2016). 17D yellow fever vaccine elicits comparable long-term immune responses in healthy individuals and immune-compromised patients. J. Infect..

[67] Reinhardt B., Jaspert R., Niedrig M., Kostner C., L’age-Stehr J. (1998). Development of viremia and humoral and cellular parameters of immune activation after vaccination with yellow fever virus strain 17D: A model of human flavivirus infection. J. Med. Virol..

[68] Douam F., Soto Albrecht Y.E., Hrebikova G., Sadimin E., Davidson C., Kotenko S.V., Ploss A. (2017). Type III Interferon-Mediated Signaling Is Critical for Controlling Live Attenuated Yellow Fever Virus Infection In Vivo. mBio.

[69] Lam L.M., Watson A.M., Ryman K.D., Klimstra W.B. (2018). Gamma-interferon exerts a critical early restriction on replication and dissemination of yellow fever virus vaccine strain 17D-204. NPJ Vaccines.

[70] Lindsey N.P., Horiuchi K.A., Fulton C., Panella A.J., Kosoy O.I., Velez J.O., Krow-Lucal E.R., Fischer M., Staples J.E. (2018). Persistence of yellow fever virus-specific neutralizing antibodies after vaccination among US travellers. J. Travel. Med..

[71] Stryhn A., Kongsgaard M., Rasmussen M., Harndahl M.N., Osterbye T., Bassi M.R., Thybo S., Gabriel M., Hansen M.B., Nielsen M. (2020). A Systematic, Unbiased Mapping of CD8(+) and CD4(+) T Cell Epitopes in Yellow Fever Vaccinees. Front. Immunol..

[72] James E.A., LaFond R.E., Gates T.J., Mai D.T., Malhotra U., Kwok W.W. (2013). Yellow fever vaccination elicits broad functional CD4+ T cell responses that recognize structural and nonstructural proteins. J. Virol..

[73] Wec A.Z., Haslwanter D., Abdiche Y.N., Shehata L., Pedreno-Lopez N., Moyer C.L., Bornholdt Z.A., Lilov A., Nett J.H., Jangra R.K. (2020). Longitudinal dynamics of the human B cell response to the yellow fever 17D vaccine. Proc. Natl. Acad. Sci. USA.

[74] Maciel M., Cruz Fda S., Cordeiro M.T., da Motta M.A., Cassemiro K.M., Maia Rde C., de Figueiredo R.C., Galler R., Freire Mda S., August J.T. (2015). A DNA vaccine against yellow fever virus: Development and evaluation. PLoS Negl. Trop. Dis..

[75] Querec T.D., Akondy R.S., Lee E.K., Cao W., Nakaya H.I., Teuwen D., Pirani A., Gernert K., Deng J., Marzolf B. (2009). Systems biology approach predicts immunogenicity of the yellow fever vaccine in humans. Nat. Immunol..

[76] Miller J.D., van der Most R.G., Akondy R.S., Glidewell J.T., Albott S., Masopust D., Murali-Krishna K., Mahar P.L., Edupuganti S., Lalor S. (2008). Human effector and memory CD8+ T cell responses to smallpox and yellow fever vaccines. Immunity.

[77] Akondy R.S., Fitch M., Edupuganti S., Yang S., Kissick H.T., Li K.W., Youngblood B.A., Abdelsamed H.A., McGuire D.J., Cohen K.W. (2017). Origin and differentiation of human memory CD8 T cells after vaccination. Nature.

[78] Wrammert J., Miller J., Akondy R., Ahmed R. (2009). Human immune memory to yellow fever and smallpox vaccination. J. Clin. Immunol..

[79] Ahmed R., Akondy R.S. (2011). Insights into human CD8(+) T-cell memory using the yellow fever and smallpox vaccines. Immunol. Cell Biol..

[80] Piras-Douce F., Broudic K., Chautard E., Raynal F., Courtois V., Gautheron S., Mantel N. (2023). Evaluation of safety and immuno-efficacy of a next generation live-attenuated yellow fever vaccine in cynomolgus macaques. Vaccine.

[81] Fuertes Marraco S.A., Soneson C., Cagnon L., Gannon P.O., Allard M., Maillard S.A., Montandon N., Rufer N., Waldvogel S., Delorenzi M. (2015). Long-lasting stem cell–like memory CD8+ T cells with a naïve-like profile upon yellow fever vaccination. Sci. Transl. Med..

[82] Kling K., Domingo C., Bogdan C., Duffy S., Harder T., Howick J., Kleijnen J., McDermott K., Wichmann O., Wilder-Smith A. (2022). Duration of Protection After Vaccination Against Yellow Fever: A Systematic Review and Meta-Analysis. Clin. Infect. Dis..

[83] Vaccines C.G.f.S.o.Y.F. (2019). Duration of immunity in recipients of two doses of 17DD yellow fever vaccine. Vaccine.

[84] Collaborative Group for Studies on Yellow Fever Vaccines (2014). Duration of post-vaccination immunity against yellow fever in adults. Vaccine.

[85] Wigg de Araujo Lagos L., de Jesus Lopes de Abreu A., Caetano R., Braga J.U. (2023). Yellow fever vaccine safety in immunocompromised individuals: A systematic review and meta-analysis. J. Travel. Med..

[86] Gerhardt C.M.B., Castro A., Pastorino A.C., Dorna M.B., Nunes-Santos C.J., Aquilante B.P., Miyaji K.T., Lopes M.H. (2020). Safety of yellow fever vaccine administration in confirmed egg-allergic patients. Vaccine.

[87] Ramírez-Giraldo R.H., Giraldo-Avila P.A., Calle A.M., Santamaria L.C., Sánchez J. (2025). No Yellow Fever Vaccine Reactions in IgE-Mediated Egg Allergic Patients. Int. Arch. Allergy Immunol..

[88] Sharma K., Perrett K.P., Wood N. (2020). Yellow Fever Vaccination In EGG-Allergic Children. Pediatr. Infect. Dis. J..

[89] Miller E.R., McNeil M.M., Moro P.L., Duffy J., Su J.R. (2020). The reporting sensitivity of the Vaccine Adverse Event Reporting System (VAERS) for anaphylaxis and for Guillain-Barré syndrome. Vaccine.

[90] Rojas A., Hachey W., Kaur G., Korejwo J., Muhammad R. (2023). Enhanced safety surveillance of STAMARIL^®^ yellow fever vaccine provided under the expanded access investigational new drug program in the USA. J. Travel. Med..

[91] Tanno L.K., Caminati M., Pouessel G., Senna G., Demoly P. (2023). Epidemiology of anaphylaxis: Is the trend still going up?. Curr. Opin. Allergy Clin. Immunol..

[92] Lindsey N.P., Rabe I.B., Miller E.R., Fischer M., Staples J.E. (2016). Adverse event reports following yellow fever vaccination, 2007–2013. J. Travel. Med..

[93] de Menezes Martins R., da Luz Fernandes Leal M., Homma A. (2015). Serious adverse events associated with yellow fever vaccine. Hum. Vaccines Immunother..

[94] Thomas R.E., Lorenzetti D.L., Spragins W., Jackson D., Williamson T. (2011). Reporting rates of yellow fever vaccine 17D or 17DD-associated serious adverse events in pharmacovigilance data bases: Systematic review. Curr. Drug Saf..

[95] Kelso J.M., Mootrey G.T., Tsai T.F. (1999). Anaphylaxis from yellow fever vaccine. J. Allergy Clin. Immunol..

[96] Bae H.-G., Domingo C., Tenorio A., de Ory F., Muñoz J., Weber P., Teuwen D.E., Niedrig M. (2008). Immune response during adverse events after 17D-derived yellow fever vaccination in Europe. J. Infect. Dis..

[97] Chan C.Y., Chan K.R., Chua C.J., Nur Hazirah S., Ghosh S., Ooi E.E., Low J.G. (2017). Early molecular correlates of adverse events following yellow fever vaccination. JCI Insight.

[98] Thomas R.E., Lorenzetti D.L., Spragins W., Jackson D., Williamson T. (2012). The safety of yellow fever vaccine 17D or 17DD in children, pregnant women, HIV+ individuals, and older persons: Systematic review. Am. J. Trop. Med. Hyg..

[99] Vasconcelos P.F.C., Luna E.J., Galler R., Silva L.J., Coimbra T.L., Barros V.L.R.S., Monath T.P., Rodigues S.G., Laval C., Costa Z.G. (2001). Serious adverse events associated with yellow fever 17DD vaccine in Brazil: A report of two cases. Lancet.

[100] Nordin J.D., Parker E.D., Vazquez-Benitez G., Kharbanda E.O., Naleway A., Marcy S.M., Molitor B., Kuckler L., Baggs J. (2013). Safety of the yellow fever vaccine: A retrospective study. J. Travel. Med..

[101] Martins R.d.M., Maia M.d.L.d.S., Santos E.M.d., Cruz R.L.d.S., dos Santos P.R.G., Carvalho S.M.D., Sato H.K., Schermann M.T., Mohrdieck R., Leal M.d.L.F. (2010). Yellow Fever Vaccine Post-marketing Surveillance in Brazil. Procedia Vaccinol..

[102] Thomas R.E., Lorenzetti D.L., Spragins W., Jackson D., Williamson T. (2011). Active and passive surveillance of yellow fever vaccine 17D or 17DD-associated serious adverse events: Systematic review. Vaccine.

[103] Belmusto-Worn V.E., Sanchez J.L., McCARTHY K., Nichols R., Bautista C.T., Magill A.J., Pastor-Cauna G., Echevarria C., Laguna-Torres V.A., Samame B.K. (2005). Randomized, double-blind, phase III, pivotal field trial of the comparative immunogenicity, safety, and tolerability of two yellow fever 17D vaccines (ARILVAXTM and YF-VAX (R)) in healthy infants and. Am. J. Trop. Med. Hyg..

[104] de Abreu A.J.L., Cavalcante J.R., de Araújo Lagos L.W., Caetano R., Braga J.U. (2022). A Systematic Review and a Meta-Analysis of the Yellow Fever Vaccine in the Elderly Population. Vaccines.

[105] Farnsworth M.G., Khanipov K., Botnar K., Weaver S.C., Barrett A.D.T., Golovko G. (2025). Real-world evidence of yellow Fever vaccination data-driven study. Vaccine.

[106] Ledlie S., Ricci C., Pan C., Rojas A., Khromava A., Li L. (2022). Yellow fever vaccine usage in the United States and risk of neurotropic and viscerotropic disease: A retrospective cohort study using three healthcare databases. Vaccine.

[107] Avelino-Silva V.I., Miyaji K.T., Mathias A., Costa D.A., de Carvalho Dias J.Z., Lima S.B., Simoes M., Freire M.S., Caiaffa-Filho H.H., Hong M.A. (2016). CD4/CD8 Ratio Predicts Yellow Fever Vaccine-Induced Antibody Titers in Virologically Suppressed HIV-Infected Patients. J. Acquir. Immune Defic. Syndr..

[108] Bovay A., Nassiri S., Maby-El Hajjami H., Marcos Mondéjar P., Akondy R.S., Ahmed R., Lawson B., Speiser D.E., Fuertes Marraco S.A. (2020). Minimal immune response to booster vaccination against Yellow Fever associated with pre-existing antibodies. Vaccine.

[109] Kongsgaard M., Bassi M.R., Rasmussen M., Skjødt K., Thybo S., Gabriel M., Hansen M.B., Christensen J.P., Thomsen A.R., Buus S. (2017). Adaptive immune responses to booster vaccination against yellow fever virus are much reduced compared to those after primary vaccination. Sci. Rep..

[110] Lecomte E., Laureys G., Verbeke F., Domingo Carrasco C., Van Esbroeck M., Huits R. (2020). A clinician’s perspective on yellow fever vaccine-associated neurotropic disease. J. Travel. Med..

[111] McMahon A.W., Eidex R.B., Marfin A.A., Russell M., Sejvar J.J., Markoff L., Hayes E.B., Chen R.T., Ball R., Braun M.M. (2007). Neurologic disease associated with 17D-204 yellow fever vaccination: A report of 15 cases. Vaccine.

[112] de Andrade Gandolfi F., Estofolete C.F., Wakai M.C., Negri A.F., Barcelos M.D., Vasilakis N., Nogueira M.L. (2023). Yellow Fever Vaccine-Related Neurotropic Disease in Brazil Following Immunization with 17DD. Vaccines.

[113] Chen L.H., Kozarsky P.E., Visser L.G. (2019). What’s Old Is New Again: The Re-emergence of Yellow Fever in Brazil and Vaccine Shortages. Clin. Infect. Dis..

[114] Monath T.P. (2012). Review of the risks and benefits of yellow fever vaccination including some new analyses. Expert. Rev. Vaccines.

[115] Struchiner C.J., Luz P.M., Dourado I., Sato H.K., Aguiar S.G., Ribeiro J.G., Soares R.C., Codeco C.T. (2004). Risk of fatal adverse events associated with 17DD yellow fever vaccine. Epidemiol. Infect..

[116] Le Hir A., Durand G.A., Boucraut J., Garnier A., Mura M., Diamantis S., Carles M., Durand C., Schweitzer C., Audouard C. (2024). Yellow fever vaccine-associated neurologic and viscerotropic disease: A 10-year case series of the French National Reference Center for Arboviruses with clinical and immunological insights. J. Travel. Med..

[117] Leung W.S., Chan M.C., Chik S.H., Tsang T.Y. (2016). First case of yellow fever vaccine-associated viscerotropic disease (YEL-AVD) in Hong Kong. J. Travel. Med..

[118] Wang H.J., Guo Y., He M.J., Liu Z.Y., Ye Q., Huang X.Y., Deng Y.Q., Li X.F., Qin C.F. (2022). Development of a Bicistronic Yellow Fever Live Attenuated Vaccine with Reduced Neurovirulence and Viscerotropism. Microbiol. Spectr..

[119] Brunaldi M.O., Silva R.J.C., Fabro A.T., de Almeida E.A.D.C., Basile-Filho A., Auxiliadora-Martins M., Menegueti M.G., Beloddi M.I., Alves Esposito D.L., Lopes da Fonseca B.A. (2021). Case Report: Fatal Viscerotropic Disease in a Young Woman Following Yellow Fever Vaccination. Am. J. Trop. Med. Hyg..

[120] Vieira L.J.T., Goebel G.A., Barcelos Y., Cunha L.O., Santos L.T.M., Romanelli R.M.C., Minafra F.G., Carvalho A.L., Carvalho L.F.A., Diniz L.M.O. (2024). Fatal viscerotropic and neurotropic disease after yellow fever vaccine: A rare manifestation leading to diagnosis of severe combined immunodeficiency in an infant. Rev. Inst. Med. Trop..

[121] Volkov L., Grard G., Bollaert P.E., Durand G.A., Cravoisy A., Conrad M., Nace L., Courte G., Marnai R., Leparc-Goffart I. (2020). Viscerotropic disease and acute uveitis following yellow fever vaccination: A case report. BMC Infect. Dis..

[122] Chippaux J.P., Chippaux A. (2018). Yellow fever in Africa and the Americas: A historical and epidemiological perspective. J. Venom. Anim. Toxins Incl. Trop. Dis..

[123] Chen L.H., Wilson M.E. (2020). Yellow fever control: Current epidemiology and vaccination strategies. Trop. Dis. Travel. Med. Vaccines.

[124] de Lima R.C., da Costa Faria N.R., de Carvalho A.T. (2025). Flow Cytometry as Immunoassay Tool for Research on Yellow Fever Virus. Methods Mol. Biol..

[125] Nomhwange T., Jean Baptiste A.E., Ezebilo O., Oteri J., Olajide L., Emelife K., Hassan S., Nomhwange E.R., Adejoh K., Ireye F. (2021). The resurgence of yellow fever outbreaks in Nigeria: A 2-year review 2017–2019. BMC Infect Dis.

[126] Diagne M.M., Ndione M.H.D., Gaye A., Barry M.A., Diallo D., Diallo A., Mwakibete L.L., Diop M., Ndiaye E.H., Ahyong V. (2021). Yellow Fever Outbreak in Eastern Senegal, 2020–2021. Viruses.

[127] Salomon O.D., Arias A.R. (2022). The second coming of urban yellow fever in the Americas: Looking the past to see the future. An. Acad. Bras. Cienc..

[128] Rodriguez-Morales A.J., Chang-Cheng B., Gross R., Llanque-Espinoza O.E., Villamil-Macareno J., Pacheco-Jimenez C., Pineda-Bersoza G.B., Delgado-Torres N.F., Sanchez-Rojas I.C., Solarte-Jimenez C.L. (2025). Clinical features of yellow fever in cases from Bolivia, Ecuador, Colombia, and Peru (2023–2025): A descriptive retrospective study. New Microbes New Infect..

[129] Zhao S., Stone L., Gao D., He D. (2018). Modelling the large-scale yellow fever outbreak in Luanda, Angola, and the impact of vaccination. PLoS Negl. Trop. Dis..

[130] Mensah E.A., Gyasi S.O., Nsubuga F., Alali W.Q. (2024). A proposed One Health approach to control yellow fever outbreaks in Uganda. One Health Outlook.

[131] Ortiz-Martínez Y., Patiño-Barbosa A.M., Rodriguez-Morales A.J. (2017). Yellow fever in the Americas: The growing concern about new epidemics. F1000Research.

[132] Cunha M.d.P., Duarte-Neto A.N., Pour S.Z., Ortiz-Baez A.S., Černý J., Pereira B.B.d.S., Braconi C.T., Ho Y.-L., Perondi B., Sztajnbok J. (2019). Origin of the São Paulo Yellow Fever epidemic of 2017–2018 revealed through molecular epidemiological analysis of fatal cases. Sci. Rep..

[133] Tomori O. (2004). Yellow fever: The recurring plague. Crit. Rev. Clin. Lab. Sci..

[134] Faria N.R., Kraemer M.U., Hill S.C., Góes de Jesus J., Aguiar R.d., Iani F.C., Xavier J., Quick J., du Plessis L., Dellicour S. (2018). Genomic and epidemiological monitoring of yellow fever virus transmission potential. Science.

[135] do Carmo Cupertino M., Garcia R., Gomes A.P., de Paula S.O., Mayers N., Siqueira-Batista R. (2019). Epidemiological, prevention and control updates of yellow fever outbreak in Brazil. Asian Pac. J. Trop. Med..

[136] Collins N.D., Barrett A.D. (2017). Live Attenuated Yellow Fever 17D Vaccine: A Legacy Vaccine Still Controlling Outbreaks In Modern Day. Curr. Infect. Dis. Rep..

[137] Kraemer M.U., Faria N.R., Reiner R.C., Golding N., Nikolay B., Stasse S., Johansson M.A., Salje H., Faye O., Wint G.W. (2017). Spread of yellow fever virus outbreak in Angola and the Democratic Republic of the Congo 2015–16: A modelling study. Lancet Infect. Dis..

[138] Thomas C., Michaud C., Gaillet M., Carrión-Nessi F.S., Forero-Peña D.A., Lacerda M.V.G., Duchemin J.-B., Rodovalho S., Vreden S., Ramos R. (2023). Yellow Fever Reemergence Risk in the Guiana Shield: A Comprehensive Review of Cases Between 1990 and 2022. Curr. Trop. Med. Rep..

[139] Wasserman S., Tambyah P.A., Lim P.L. (2016). Yellow fever cases in Asia: Primed for an epidemic. Int. J. Infect. Dis..

[140] Ndeffo-Mbah M.L., Pandey A. (2020). Global Risk and Elimination of Yellow Fever Epidemics. J. Infect. Dis..

[141] Jean K., Hamlet A., Benzler J., Cibrelus L., Gaythorpe K.A., Sall A., Ferguson N.M., Garske T. (2020). Eliminating yellow fever epidemics in Africa: Vaccine demand forecast and impact modelling. PLoS Negl. Trop. Dis..

[142] Silva T., Nogueira de Sa A., Prates E.J.S., Rodrigues D.E., Silva T., Matozinhos F.P., Vieira E.W.R. (2022). Yellow fever vaccination before and during the covid-19 pandemic in Brazil. Rev. Saude Publica.

[143] Casey R.M., Harris J.B., Ahuka-Mundeke S., Dixon M.G., Kizito G.M., Nsele P.M., Umutesi G., Laven J., Kosoy O., Paluku G. (2019). Immunogenicity of Fractional-Dose Vaccine during a Yellow Fever Outbreak—Final Report. N. Engl. J. Med..

[144] Doshi R.H., Mukadi P.K., Casey R.M., Kizito G.M., Gao H., Nguete U.B., Laven J., Sabi L., Kaba D.K., Muyembe-Tamfum J.J. (2024). Immunological response to fractional-dose yellow fever vaccine administered during an outbreak in Kinshasa, Democratic Republic of the Congo: Results 5 years after vaccination from a prospective cohort study. Lancet Infect. Dis..

[145] Nnaji C.A., Shey M.S., Adetokunboh O.O., Wiysonge C.S. (2020). Immunogenicity and safety of fractional dose yellow fever vaccination: A systematic review and meta-analysis. Vaccine.

[146] Vannice K., Wilder-Smith A., Hombach J. (2018). Fractional-Dose Yellow Fever Vaccination—Advancing the Evidence Base. N. Engl. J. Med..

[147] Rodriguez-Morales A.J., Torres-Hernández D., Guevara M.E., Chang-Cojulun A., Brea-Del Castillo J., Rios-Blanco R., Mérida-Barrios M.I., Palmieri M., Avila-Agüero M.L. (2025). Yellow fever in children and adolescents amid the South American outbreak, 2024/2025. New Microbes New Infect.

[148] Roukens A.H.E., Visser L.G. (2019). Fractional-dose yellow fever vaccination: An expert review. J. Travel. Med..

[149] Manikandan S., Mathivanan A., Bora B., Hemaladkshmi P., Abhisubesh V., Poopathi S. (2023). A review on vector borne disease transmission: Current strategies of mosquito vector control. Indian. J. Entomol..

[150] Kleinert R.D.V., Montoya-Diaz E., Khera T., Welsch K., Tegtmeyer B., Hoehl S., Ciesek S., Brown R.J.P. (2019). Yellow Fever: Integrating Current Knowledge with Technological Innovations to Identify Strategies for Controlling a Re-Emerging Virus. Viruses.

[151] Garske T., Van Kerkhove M.D., Yactayo S., Ronveaux O., Lewis R.F., Staples J.E., Perea W., Ferguson N.M. (2014). Yellow Fever Expert Committee. Yellow Fever in Africa: Estimating the burden of disease and impact of mass vaccination from outbreak and serological data. PLoS Med..

[152] Raimundo S.M., Yang H.M., Massad E. (2016). Modeling Vaccine Preventable Vector-Borne Infections: Yellow Fever as a Case Study. J. Biol. Syst..

[153] Tyagi P., Ganguly M., Manney S., Wadkar K., Ingle N., Gairola S., Dhere R., Lapini G., Cantaloni P. (2025). Plaque reduction neutralization test (PRNT_50_) for the detection of anti-yellow fever antibodies from clinical samples. Vaccine.

[154] Condit R.C., Kim D., Robertson J.S., Excler J.L., Gurwith M., Monath T.P., Pavlakis G., Fast P.E., Smith J., Smith E.R. (2020). The Brighton Collaboration standardized template for collection of key information for benefit-risk assessment of viral vector vaccines. Vaccine.

[155] Davis E.H., Barrett A.D.T. (2020). Structure-Function of the Yellow Fever Virus Envelope Protein: Analysis of Antibody Epitopes. Viral Immunol..

[156] Davis E.H., Beck A.S., Strother A.E., Thompson J.K., Widen S.G., Higgs S., Wood T.G., Barrett A.D. (2019). Attenuation of Live-Attenuated Yellow Fever 17D Vaccine Virus Is Localized to a High-Fidelity Replication Complex. mBio.

[157] Fernandez-Garcia M.D., Meertens L., Chazal M., Hafirassou M.L., Dejarnac O., Zamborlini A., Despres P., Sauvonnet N., Arenzana-Seisdedos F., Jouvenet N. (2016). Vaccine and Wild-Type Strains of Yellow Fever Virus Engage Distinct Entry Mechanisms and Differentially Stimulate Antiviral Immune Responses. mBio.

[158] Koblischke M., Mackroth M.S., Schwaiger J., Fae I., Fischer G., Stiasny K., Heinz F.X., Aberle J.H. (2017). Protein structure shapes immunodominance in the CD4 T cell response to yellow fever vaccination. Sci. Rep..

[159] Moore J., Ahmed H., Jia J., Akondy R., Ahmed R., Antia R. (2018). What Controls the Acute Viral Infection Following Yellow Fever Vaccination?. Bull. Math. Biol..

[160] Medina-Magues L.G., Muhe J., Jasny E., Medina-Magues E.S., Roth N., Lopera-Madrid J., Salas-Quinchucua C., Knuese C., Petsch B., Osorio J.E. (2023). Immunogenicity and protective activity of mRNA vaccine candidates against yellow fever virus in animal models. NPJ Vaccines.

[161] Monath T.P., Seligman S.J., Robertson J.S., Guy B., Hayes E.B., Condit R.C., Excler J.L., Mac L.M., Carbery B., Chen R.T. (2015). Live virus vaccines based on a yellow fever vaccine backbone: Standardized template with key considerations for a risk/benefit assessment. Vaccine.

[162] Al-Halifa S., Gauthier L., Arpin D., Bourgault S., Archambault D. (2019). Nanoparticle-Based Vaccines Against Respiratory Viruses. Front. Immunol..

[163] Ghattas M., Dwivedi G., Lavertu M., Alameh M.G. (2021). Vaccine Technologies and Platforms for Infectious Diseases: Current Progress, Challenges, and Opportunities. Vaccines.

[164] Kitui S.K., Juma E., Ndalama M.T., Chilot D., Tolossa D., Woldemedhin B., Muzazu S.G.Y., Digamo K., Mungania J., Manyazewal T. (2025). Trends in uptake and impact of thermostable vaccines in Africa. Ther. Adv. Vaccines Immunother..

[165] Hansen C.A., Barrett A.D.T. (2021). The Present and Future of Yellow Fever Vaccines. Pharmaceuticals.

[166] Teitelbaum P., Bui Y.G., Libman M., McCarthy A. (2018). Fractional dosing of yellow fever vaccine during shortages: Perspective from Canada. J. Travel Med..

[167] Montalvo Zurbia-Flores G., Rollier C.S., Reyes-Sandoval A. (2022). Re-thinking yellow fever vaccines: Fighting old foes with new generation vaccines. Hum. Vaccin. Immunother..

[168] Yan K., Vet L.J., Tang B., Hobson-Peters J., Rawle D.J., Le T.T., Larcher T., Hall R.A., Suhrbier A. (2020). A Yellow Fever Virus 17D Infection and Disease Mouse Model Used to Evaluate a Chimeric Binjari-Yellow Fever Virus Vaccine. Vaccines.

[169] Abbo S.R., Yan K., Geertsema C., Hick T.A.H., Altenburg J.J., Nowee G., van Toor C., van Lent J.W., Nakayama E., Tang B. (2025). Virus-like particle vaccine with authentic quaternary epitopes protects against Zika virus-induced viremia and testicular damage. J. Virol..

[170] Amanna I.J., Thomas A., Engelmann F., Hammarlund E., Raué H.P., Bailey A.L., Poore E.A., Quintel B.K., Lewis A.D., Axthelm M.K. (2024). Development of a hydrogen peroxide-inactivated vaccine that protects against viscerotropic yellow fever in a non-human primate model. Cell Rep. Med..

[171] Oreshkova N., Myeni S.K., Mishra N., Albulescu I.C., Dalebout T.J., Snijder E.J., Bredenbeek P.J., Dallmeier K., Kikkert M. (2021). A Yellow Fever 17D Virus Replicon-Based Vaccine Platform for Emerging Coronaviruses. Vaccines.

[172] Fonseca J.A., McCaffery J.N., Caceres J., Kashentseva E., Singh B., Dmitriev I.P., Curiel D.T., Moreno A. (2018). Inclusion of the murine IgGκ signal peptide increases the cellular immunogenicity of a simian adenoviral vectored Plasmodium vivax multistage vaccine. Vaccine.

[173] Kardani K., Bolhassani A., Shahbazi S. (2016). Prime-boost vaccine strategy against viral infections: Mechanisms and benefits. Vaccine.

[174] Levine M.Z., Holiday C., Jefferson S., Gross F.L., Liu F., Li S., Friel D., Boutet P., Innis B.L., Mallett C.P. (2019). Heterologous prime-boost with A(H5N1) pandemic influenza vaccines induces broader cross-clade antibody responses than homologous prime-boost. NPJ Vaccines.

[175] Pasin C., Balelli I., Van Effelterre T., Bockstal V., Solforosi L., Prague M., Douoguih M., Thiébaut R. (2019). Dynamics of the Humoral Immune Response to a Prime-Boost Ebola Vaccine: Quantification and Sources of Variation. J. Virol..

[176] Santos-Peral A., Luppa F., Goresch S., Nikolova E., Zaucha M., Lehmann L., Dahlstroem F., Karimzadeh H., Thorn-Seshold J., Winheim E. (2024). Prior flavivirus immunity skews the yellow fever vaccine response to cross-reactive antibodies with potential to enhance dengue virus infection. Nat. Commun..

[177] Watson A.M., Klimstra W.B. (2017). T Cell-Mediated Immunity towards Yellow Fever Virus and Useful Animal Models. Viruses.

[178] Servadio J.L., Munoz-Zanzi C., Convertino M. (2022). Environmental determinants predicting population vulnerability to high yellow fever incidence. R. Soc. Open Sci..

[179] Adrien N., Hyde T.B., Gacic-Dobo M., Hombach J., Krishnaswamy A., Lambach P. (2019). Differences between coverage of yellow fever vaccine and the first dose of measles-containing vaccine: A desk review of global data sources. Vaccine.

[180] Wu J.T., Peak C.M., Leung G.M., Lipsitch M. (2016). Fractional dosing of yellow fever vaccine to extend supply: A modelling study. Lancet.

[181] Gubler D.J., Almeida M.A.B., Cardoso J.d.C., dos Santos E., da Fonseca D.F., Cruz L.L., Faraco F.J.C., Bercini M.A., Vettorello K.C., Porto M.A. (2014). Surveillance for Yellow Fever Virus in Non-Human Primates in Southern Brazil, 2001–2011: A Tool for Prioritizing Human Populations for Vaccination. PLoS Neglected Trop. Dis..

[182] Selemane I. (2019). Epidemiological monitoring of the last outbreak of yellow fever in Brazil—An outlook from Portugal. Travel. Med. Infect. Dis..

[183] Andrade M.S., Campos F.S., Oliveira C.H., Oliveira R.S., Campos A.A.S., Almeida M.A.B., Fonseca V.S., Simonini-Teixeira D., Sevá A.D.P., Temponi A.O.D. (2022). Fast surveillance response reveals the introduction of a new yellow fever virus sub-lineage in 2021, in Minas Gerais, Brazil. Mem. Inst. Oswaldo Cruz.

[184] Hyde T.B., Andrus J.K., Dietz V.J., Integrated All V.P.D.S.W.G., Andrus J.K., Hyde T.B., Lee C.E., Widdowson M.A., Verani J.R., Friedman C. (2013). Critical issues in implementing a national integrated all-vaccine preventable disease surveillance system. Vaccine.

[185] Zhao Y., Zhang X., Shu S., Sun Y., Feng X., Zhang S. (2018). Yellow Fever: A Re-Emerging Threat. Health.

[186] Cruz A.C.R., Hernandez L.H.A., Aragao C.F., da Paz T.Y.B., da Silva S.P., da Silva F.S., de Aquino A.A., Cereja G., Nascimento B., Rosa Junior J.W. (2023). The Importance of Entomo-Virological Investigation of Yellow Fever Virus to Strengthen Surveillance in Brazil. Trop. Med. Infect. Dis..

[187] Aliaga-Samanez A., Real R., Segura M., Marfil-Daza C., Olivero J. (2022). Yellow fever surveillance suggests zoonotic and anthroponotic emergent potential. Commun. Biol..

[188] Mantilla-Granados J.S., Sarmiento-Senior D., Manzano J., Calderon-Pelaez M.A., Velandia-Romero M.L., Buitrago L.S., Castellanos J.E., Olano V.A. (2022). Multidisciplinary approach for surveillance and risk identification of yellow fever and other arboviruses in Colombia. One Health.

[189] Mangudo C., Aparicio J.P., Rossi G.C., Gleiser R.M. (2018). Tree hole mosquito species composition and relative abundances differ between urban and adjacent forest habitats in northwestern Argentina. Bull. Entomol. Res..

[190] Williams D.T., Mackenzie J.S., Bingham J. (2019). Flaviviruses. Diseases of Swine.

[191] Subramaniam K.S., Lant S., Goodwin L., Grifoni A., Weiskopf D., Turtle L. (2020). Two Is Better Than One: Evidence for T-Cell Cross-Protection Between Dengue and Zika and Implications on Vaccine Design. Front. Immunol..

[192] Zuckerman J.N., Hatz C., Kantele A. (2017). Review of current typhoid fever vaccines, cross-protection against paratyphoid fever, and the European guidelines. Expert. Rev. Vaccines.

[193] Cadenas-Fernández E., Barroso-Arévalo S., Kosowska A., Díaz-Frutos M., Gallardo C., Rodríguez-Bertos A., Bosch J., Sánchez-Vizcaíno J.M., Barasona J.A. (2024). Challenging boundaries: Is cross-protection evaluation necessary for African swine fever vaccine development? A case of oral vaccination in wild boar. Front. Immunol..

[194] Possas C., Lourenço-de-Oliveira R., Tauil P.L., Pinheiro F.d.P., Pissinatti A., Cunha R.V.d., Freire M., Martins R.M., Homma A. (2018). Yellow fever outbreak in Brazil: The puzzle of rapid viral spread and challenges for immunisation. Mem. Inst. Oswaldo Cruz.

[195] de Oliveira Figueiredo P., Stoffella-Dutra A.G., Barbosa Costa G., Silva de Oliveira J., Dourado Amaral C., Duarte Santos J., Soares Rocha K.L., Araujo Junior J.P., Lacerda Nogueira M., Zaza Borges M.A. (2020). Re-emergence of yellow fever in Brazil during 2016–2019: Challenges, lessons learned, and perspectives. Viruses.

[196] Hernandez A., Lee J., Kang H. (2025). Navigating the Interconnected Web of Health: A Comprehensive Review of the One Health Paradigm and Its Implications for Disease Management. Yonsei Med. J..

[197] Fleischmann W.A., Cao L.C., Nurjadi D., Velavan T.P. (2024). Addressing the rise of autochthonous vector-borne diseases in a warming Europe. Int. J. Infect. Dis..

[198] Kraemer M.U.G., Reiner R.C., Brady O.J., Messina J.P., Gilbert M., Pigott D.M., Yi D., Johnson K., Earl L., Marczak L.B. (2019). Past and future spread of the arbovirus vectors Aedes aegypti and Aedes albopictus. Nat. Microbiol..

[199] Cañete R., Vega-Jiménez J., Rodriguez-Morales A.J. (2025). Beyond dengue and Oropouche: The urgent need for yellow fever preparedness in Cuba. New Microbes New Infect..

[200] Visser L.G. (2019). Fractional-dose yellow fever vaccination: How much more can we do with less?. Curr. Opin. Infect. Dis..

[201] Wilke A.B.B., Farina P., Ajelli M., Canale A., Dantas-Torres F., Otranto D., Benelli G. (2025). Human migrations, anthropogenic changes, and insect-borne diseases in Latin America. Parasit. Vectors.

